# Photocatalytic dye degradation and biological activities of the Fe_2_O_3_/Cu_2_O nanocomposite†

**DOI:** 10.1039/c8ra09929d

**Published:** 2019-03-14

**Authors:** Mavinakere Ramesh Abhilash, Gangadhar Akshatha, Shivanna Srikantaswamy

**Affiliations:** Department of Studies in Environmental Science, University of Mysore Manasagangotri Mysore 570006 India; Centre for Materials Science and Technology, Vijnana Bhavan, University of Mysore Manasagangotri Mysore 570006 India abilashmrenvi@gmail.com

## Abstract

The present study reports the synthesis of the Fe_2_O_3_/Cu_2_O nanocomposite *via* a facile hydrothermal route. The products were characterized using X-ray diffractometry (XRD), Fourier-transform infrared spectroscopy (FTIR), dynamic light scattering (DLS), high-resolution transmission electron microscopy (HR-TEM), energy dispersive spectroscopy (EDS) and Brunauer–Emmett–Teller (BET) techniques. The composition, morphology and structural features of the nanoparticles were found to be size-dependent due to the temperature response in the particular time log during hydrothermal synthesis. HR-TEM confirmed the formation of hexagonal rod-shaped bare Cu_2_O, rhombohedral-shaped Fe_2_O_3_ and composite assembly. Rhodamine-B (RB) and Janus green (JG) were chosen as model dyes for the degradation studies. Photocatalytic degradation of the dyes was deliberated by altering the catalyst and dye concentrations. The results showed that the Rhodamine-B (RB) and Janus green (JG) dyes were degraded within a short time span. The synthesized materials were found to be highly stable in the visible light-driven degradation of the dyes; showed antibacterial activity against *E. coli*, *P. aeruginosa*, *Staph. aureus* and *B. subtilis*; and exhibited less toxicity against the *Musmusculus* skin melanoma cells (B16-F10). The fusion of these advantages paves the way for further applications in energy conversion, biological applications as well as in environmental remediation.

## Abstract 2

The present study reports the synthesis of the Fe_2_O_3_/Cu_2_O nanocomposite *via* a facile hydrothermal route.

## Graphical abstract

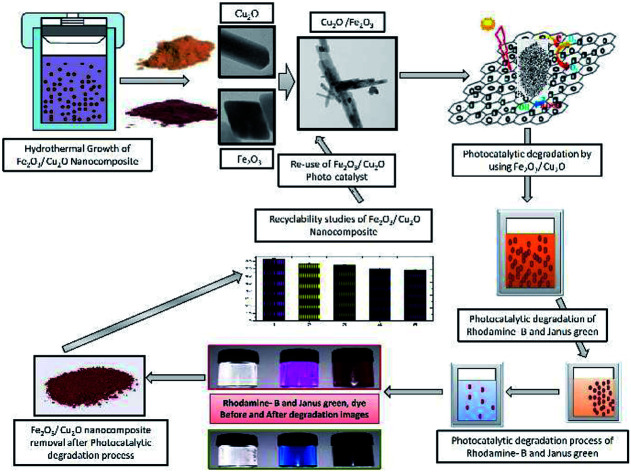

## 1 Introduction

**Fig. 1:**
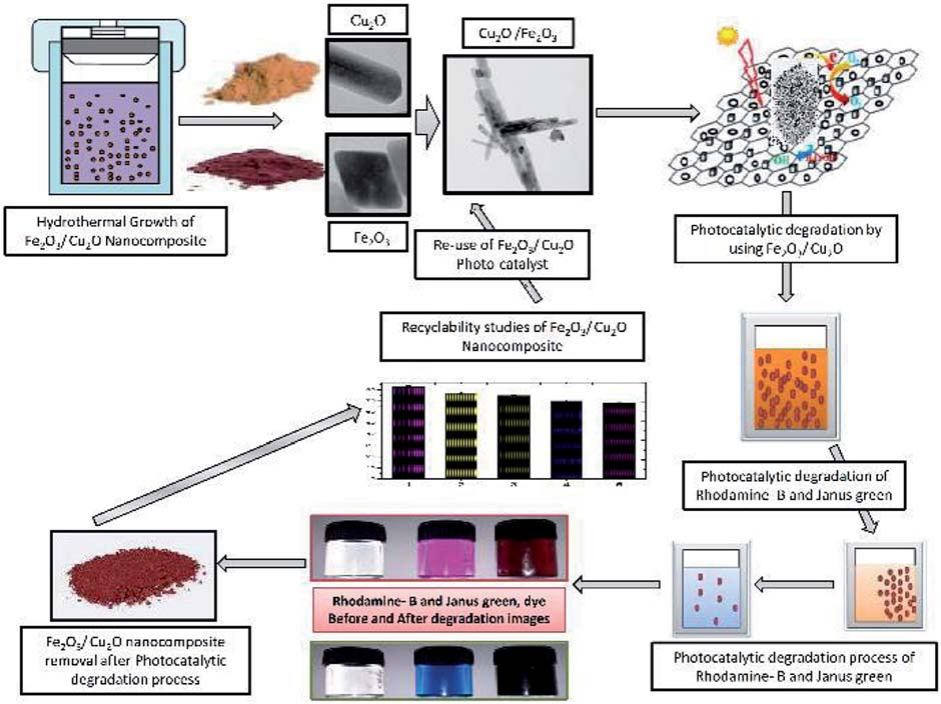
Symmetric representation of the hydrothermal growth of Fe_2_O_3_/Cu_2_O nanocomposites; the photocatalytic activity of dye degradation and recyclability.

Photocatalysis is a fascinating tool for energy conversion and environmental decontamination due to its conspicuous nature.^1^ Using metal-semiconductors for photocatalysis is an environmentally safe process that simulates the photosynthesis process to speed up chemical reactions that require or involve light.^2,3^ The visible light photocatalytic process has been implemented in semiconductor integrated circuits for harvesting solar energy.^4^ To overcome the band gap problem, metal oxides can be used as a photocatalyst, which, upon exposure to UV lights, visible lights or both, allows photo-excited electrons to be promoted to the conduction band to speed up the recombination of huge charge carriers.^5–7^ Broad band gap issues can also be overcome by introducing a hetero or homo barrier made with a combination of different metal oxides,^8^ nanowire,^9^ core–shells^9^ and nanowires,^10^ which offers superior charge transfer competence and commotion compared to a sole metal oxide semiconductor.^11–13^ This allows the competent and viable catalysts to perform under visible light irradiation, which constitutes nearly 46% of the astral band.^14^ In recent years, the investigation of semiconductors for harvesting energy in the visible spectrum has led to the progression of the invention of non-titania-based semiconducting photocatalysts for dye degradation.^15,16^ In addition, the plasmon-resonance of fine metal oxide nanoparticles (MNP's) also enhances the photocatalytic process by absorbing incident photons much more effectively. Cu_2_O has been used as a prolific p-type photo-catalyst with a through band gap in the visible range of 2.0–2.2 eV.^17–19^ However, Cu_2_O suffers from photo-wavering and photo-corrosion, which can over time cause deactivation in solar irradiation.^20^ Iron oxides (Fe_2_O_3_), on the other hand, are an extremely constant n-type semiconductor under standard ambient conditions.^21^ The valence band-edge of Fe_2_O_3_ is an outstanding catalyst for the photo-squalor of unrefined pollutants and is not only abundant in nature but also mass-producible.^22^ However, photocatalytic activity in Fe_2_O_3_ is often overwhelmed by its hole diffusion length (approximately, 10 nm) and an extremely short electron–hole recombination time.^23–25^ Most studies focus on the antibacterial activity of metal nanoparticles and its composites, including those that are against resistant strains of microorganisms. However, there is no exact mechanism for the antimicrobial action of metal oxide nanoparticles.^26^ The anti-microbial activity of metal oxide nanoparticles was enhanced with increasing surface area and volume of the nanoparticles and decreasing particle size.^27^ Inimitable properties and the assessment of interactions between nanomaterials and the biological system are essential.^28^ In recent years, the cell line toxicity of human cell lines using several synthesized metal oxide is a more fascinating topic in understanding the relationship between nanoparticle–cell interactions.^29,30^ The size, shape, and morphology of nanoparticles, play a vital role in mammalian cell culture medium, and the nanoparticle are much more toxic than the hydrothermally synthesized nanocomposite semiconductor.^31,32^ In this experiment, we focus on exploiting and utilizing some multi-tasking facile materials with high photocatalytic activity and biological applications. To evade these drawbacks, in an attempt to tackle the above issues, we have developed a cost-effective method for the synthesis of non-toxic Fe_2_O_3_ impurities on the Cu_2_O nanocomposite for various applications (Fig. 1 and 14). Fe_2_O_3_ burdened onto Cu_2_O and the nanocomposite was prepared by a facile method. The (p–n) hetero-junction nanocomposite was used for the effective degradation of organic pollutants; including Rhodamine-B (RB) and Janus green (JG), bacterial degradation and some additional biological screening applications.

## 2 Experimental

### 2.1. Hydrothermal preparation of Fe_2_O_3_

The hydrothermal technique has been found to be one of the best techniques to prepare Fe_2_O_3_ nanoparticles of the desired size with homogeneity in composition and an extreme degree of crystalline particles. The primary nanoparticles were synthesized by taking advantage of a hydrothermally enabled reaction between FeCl_3_ and NaOH. Ammonia (Aldrich, India), 10.14 g (37.5 mol) of FeCl_3_·6H_2_O and 7.45 g (37.5 mmol) of FeCl_2_·4H_2_O were dissolved into 25 ml of distilled water. 25 ml of 25% ammonia was added to the salt solution under stirring at 700 rpm for 2 min. Next, 15 ml of the mixture was put into a Teflon-lined stainless steel Morey autoclave. The autoclave was heated to 160 °C in an oven and maintained with a 12 hour reaction time. The effects of the reaction temperature on the product were investigated. Temperature plays a crucial role in the formation of a well-defined spherical product. The autoclave containing these chemicals was naturally cooled to room temperature, and the precipitates were then washed with distilled water and isolated under a magnet. The final products were dried at 60 °C and characterized. In the hydrothermal process, the following reaction takes place.

1 2FeCl_3_·6H_2_O + 2FeCl_2_·4H_2_O + 10NH_4_OH → 2Fe_2_O_3_ + 10NH_4_Cl + 14H_2_O + H_2_↑

### 2.2. Hydrothermal preparation of Cu_2_O

Cu_2_O nanocomposites were synthesized by using the reaction between copper sulfate and sodium hydroxide. 1.5 g of CuSO_4_·5H_2_O was first added into 80 ml of distilled water under mechanical stirring at 60 °C for 30 min, followed by the addition of 10 ml of NaOH aqueous solution (1 M) into the solution. After 1 minute, a pinch of anhydrous glucose (0.3 M) was quickly added and was mixed by mechanical stirring for about 30 minutes. The final solution was transferred into a Teflon-lined autoclave and hydrothermally treated at 80 °C for 5 hours. The resulting precipitate was collected, washed with distilled water followed by ethanol and vacuum dried at 60 °C for 6 hours.

2 2CuSO_4_·5H_2_O + 2NaOH → Cu_2_O + 2NaSO_4_ + 6H_2_O

### 2.3. Preparation of Fe_2_O_3_/Cu_2_O

Nanocatalysts such as Fe_2_O_3_ and Cu_2_O synthesized from the above method were used to synthesize Fe_2_O_3_/Cu_2_O nanocomposites by a simple ultra-sonication method, in which the calculated 1 : 1 molar ratio of Fe_2_O_3_ : Cu_2_O nanoparticles was sonicated in 50 ml of CHCl_3_ solution for 20 minutes. During sonication, the nanoparticles were dispersed uniformly into a composite structure. The sonicated solution was filtered as wet solids, which were dried at 80 °C for 5 hours.

### 2.4. Photocatalytic experiments of the removal of dyes

The photocatalytic behaviour of the synthesized nanomaterials was evaluated by the removal of Rhodamine-B (RB) and Janus green (JG) dyes (200 ml of aqueous solution of dyes (1 × 10^−5^ mol l^−1^)) under UV light radiation. The light source used was a 150 W Xe (Xenon) lamp, and the distance between the UV source and the photo-reaction vessel was 10 cm. Prior to irradiation, the suspensions were magnetically stirred in the dark for 30 min. Then, the photoreaction vessel was exposed to UV irradiation under standard ambient conditions. The selected dyes were used in conjunction with 10 mg of catalyst nanoparticles in the photo-removal experiment. At regular time intervals, 3 ml of the suspension was taken for centrifugation to separate the photocatalyst and for further evaluation using a UV-Vis absorption spectrometer. The photo-removal efficiency percentage was calculated from the equation given below. Afterward, recycling experiments were carried out for five repeated cycles to inspect the permanence of the photocatalytic Fe_2_O_3_/Cu_2_O nanocomposite. The composite catalyst was centrifuged, washed with ethanol and deionised water, and was dried before reuse for the next trial. The photo-removal efficiency percentage was calculated from the equation given below;% Photo-removal efficiency = *C*_0_ − *C*/*C*_0_ × 100where *C*_0_ is the initial concentration of dye and *C* is the concentration of dye after photo-irradiation (final).

### 2.5. Determination of antibacterial activity and live and dead cell analysis

The Fe_2_O_3_, Cu_2_O and the Fe_2_O_3_/Cu_2_O nanocomposite underwent bacterial screening along with the control by a disc diffusion method,^33–35^ against Gram-positive *Bacillus subtilis*, (MTCC 121) and *Staphylococcus aureus* (MTCC 7443) and Gram-negative *Escherichia coli*, (MTCC 7410) and *Pseudomonas aeruginosa* sp., (MTCC 733) bacteria. Hydrothermally synthesized Fe_2_O_3_, Cu_2_O and Fe_2_O_3_/Cu_2_O and the control were prepared in sterile distilled water (stock solution) over a range of different concentrations in 100 mg ml^−1^. The analysis was performed to distinguish dead and viable bacterial cells upon treatment with Fe_2_O_3_, Cu_2_O and Fe_2_O_3_/Cu_2_O NPs according to earlier reports,^36^ with minor modifications.

### 2.6. Cell culture and treatment

*Musmelanoma* cells (B16-F10) were seeded in tissue culture flasks and full-grown in Dulbecco's modified Eagle's medium (DMEM; Thermo Fisher, USA Gibco), balanced with 5% fetal bovine serum (FBS, Thermo Fisher; Gibco) and 1% penicillin/streptomycin blend (Santa Cruz Biotechnology, USA). A detailed experiment procedure was clearly provided in S2 in ESI.†

### 2.7. Statistical analysis

Systematic data replicates (three) were analyzed for every attempt and for every analysis of discrepancy (ANOVA) using SPSS-Inc. 16.0. Trivial effects of the treatments were resolved by *F* values (*p* ≤ 0.05).

## 3 Results and discussions

### 3.1. Characterization results of the synthesized Fe_2_O_3_/Cu_2_O nanocomposite

**Fig. 2:**
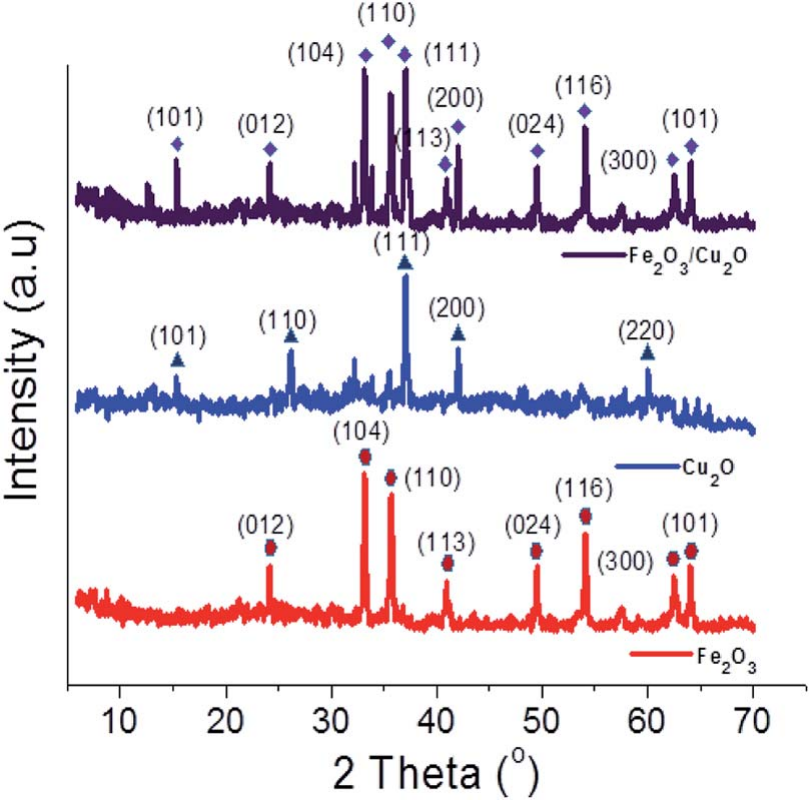
X-ray diffraction profile of Fe_2_O_3_, Cu_2_O and Fe_2_O_3_/Cu_2_O.

The XRD spectrum of the Fe_2_O_3_/Cu_2_O photocatalyst with JCPDS data for Cu_2_O and Fe_2_O_3_ are shown in (Fig. 2). The prominent diffraction peaks at the angle 2*θ* = 29.55, 36.41, 42.29, 52.4, 61.34 and 73.53° respectively correspond to the (110), (111), (200), (211), (220) and (311) crystal planes of the hexagonal rod-shaped Cu_2_O (05-0667-JCPDS). The (111) crystal plane of Cu_2_O in the composite is predicted to yield better performance in terms of photocatalytic activity.^37^ The two peaks found at 2*θ* = 33.15 and 35.61 correspond to the (104) and (110) planes of rhombohedral Fe_2_O_3_, respectively (33-0664-JCPDS).^38^ The crystalline phase of the nanocomposite Fe_2_O_3_/Cu_2_O photocatalyst suggests that the hexagonal rod-shaped Cu_2_O with rhombohedral-shaped Fe_2_O_3_ would be evenly distributed and agglomerated over the entire surface. However, the photocatalyst Fe_2_O_3_/Cu_2_O exhibited a different structure with a different crystalline phase. The HR-TEM results matched with the XRD patterns, confirming that the experimental processes were ample.

**Fig. 3:**
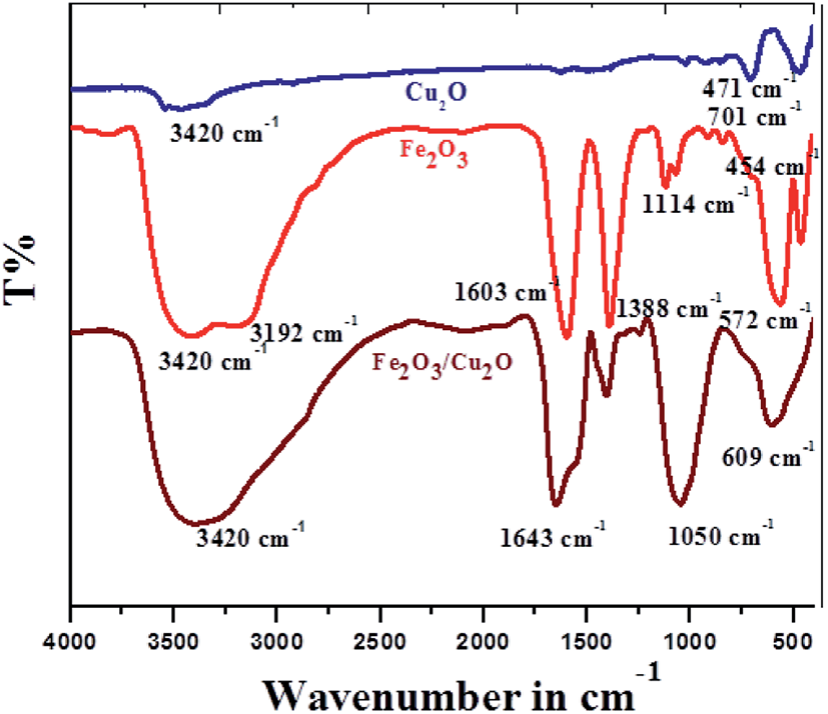
FT-IR spectrum of different Cu_2_O, Fe_2_O_3_ and Fe_2_O_3_/Cu_2_O photocatalysts.

FT-IR spectrum of the Cu_2_O, Fe_2_O_3_ and Fe_2_O_3_/Cu_2_O photocatalysts are shown in (Fig. 3). The nanocatalyst with Cu_2_O and Fe_2_O_3_ are respectively associated with the O–H bond stretching and bending vibration modes.^39^ The characteristic broad absorption peak adjusted from 700 cm^−1^ to 400 cm^−1^ as assigned to the fingerprint of the Fe_2_O_3_/Cu_2_O is 701 cm^−1^ in the Cu_2_O nanocomposite, allocated to the infrared active mode of Cu_2_O^40^ and the vigorous mode of Cu_2_O.^41^ For bare Fe_2_O_3_, the absorption peaks at 572 cm^−1^ and 454 cm^−1^ correspond to the photocatalysis reaction of Fe^3+^. FT-IR spectra of Cu_2_O, Fe_2_O_3_, and the Fe_2_O_3_/Cu_2_O composite; O^2−^ bond stretching in the FeO_6_ octahedron; and Fe^3+^ and O^2−^ bond stretching in the FeO_4_ tetrahedron, respectively, are shown.^42^ Fe_2_O_3_/Cu_2_O shows the presence of IR peaks caused by both photocatalysts, Cu_2_O and Fe_2_O_3_.

**Fig. 4:**
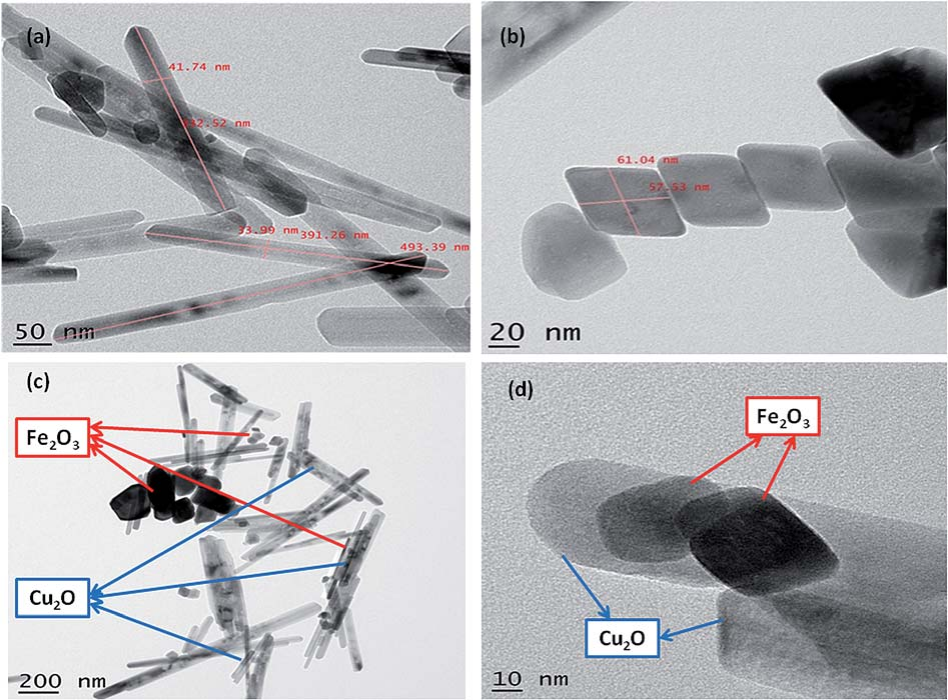
HR-TEM of (a) Cu_2_O nanoparticles, (b) Fe_2_O_3_ nanoparticles, (c) hexagonal rod-shaped Cu_2_O and rhombohedral-shaped Fe_2_O_3_; (d) Cu_2_O/Fe_2_O_3_ nanocomposite assembly, scale bar is in nm.

**Fig. 5:**
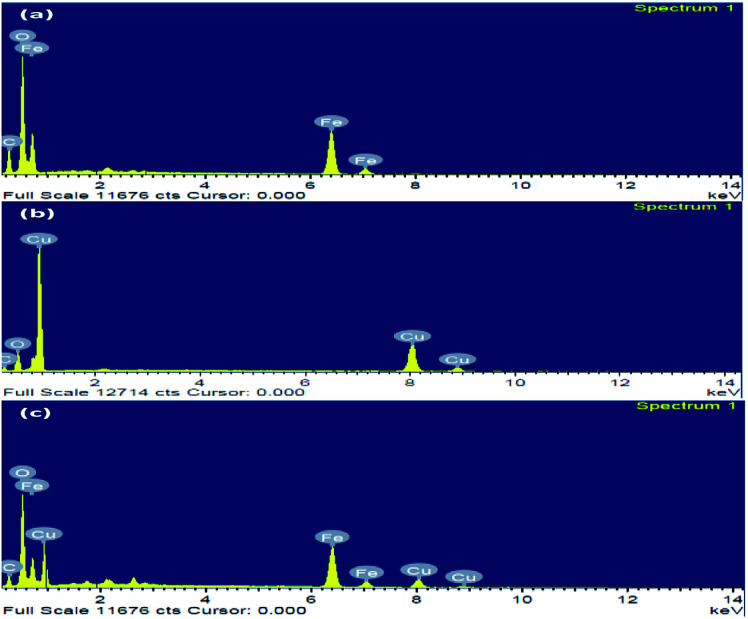
The elemental composition in the EDS of (a) Fe_2_O_3_, (b) Cu_2_O, and (c) Fe_2_O_3_/Cu_2_O.

The nanoparticles synthesized by hydrothermal method resulting in the formation of nano/mesopore structure show excellent photocatalytic performance.^43,44^ HR-TEM of the synthesized nanoparticles was shown in (Fig. 4); the high-quality images of the hexagonal rod-shaped Cu_2_O, rhombohedral-shaped Fe_2_O_3_ and Fe_2_O_3_/Cu_2_O composite nanoparticles with partial agglomeration are shown. Additionally, the nanocomposite results in the formation of mesopores between the particles, which were clearly confirmed by BET surface area and pore volume measurements. The sizes of both individual nanoparticles were between 30–60 nm, which are in accordance with the results of XRD. It was also observed that the *d*-spacing measurements of the twin domains were measured to be approximately 0.2 Å to 0.4 Å nm and corresponded to the (111) and (220) planes of Fe_2_O_3_ and Cu_2_O, respectively, and the SAED patterns confirmed the compact arrangement of the nanoparticles (Fig. S3 in ESI†). Energy dispersive spectroscopy (EDS) analysis confirmed the phase transparency of the Fe_2_O_3_, Cu_2_O and Fe_2_O_3_/Cu_2_O photocatalysts, as shown in (Fig. 5). The characteristic peaks of Fe, Cu, and O appear in the spectrum of Fe_2_O_3_/Cu_2_O and confirm its successive formation and purity (Fig. S2 in ESI†). The carbon peak appears due to the carbon tape used sample holder.

The particle size distribution was analyzed using a dynamic light scattering instrument. The Fe_2_O_3_/Cu_2_O particles were dispersed in a solvent. The distribution of particle sizes when immersed in the solvent ranged from 42–593 nm, with approximately 266 nm being the mean particle size. Histogram of the DLS analysis for the particle size distribution of Fe_2_O_3_/Cu_2_O composites is depicted in (Fig. S1 in ESI†). Zeta potential measurements by DLS were considered as an authentic technique for evaluating the particle size and zeta potentials of nanoparticles in a suspension and also plays a crucial role during interaction with other biological systems as well as environmental degradation. The particles examined presently possessed zeta potentials of −0.1 mV for Cu_2_O, 13.9 mV for Fe_2_O_3_, and −46.2 mV for the Fe_2_O_3_/Cu_2_O composite. Nanoparticles with a zeta potentials between −10 and +10 mV have a neutral charge, while if it is greater than +30 mV or less than −30 mV, it is considered to be strongly cationic or anionic.

**Fig. 6:**
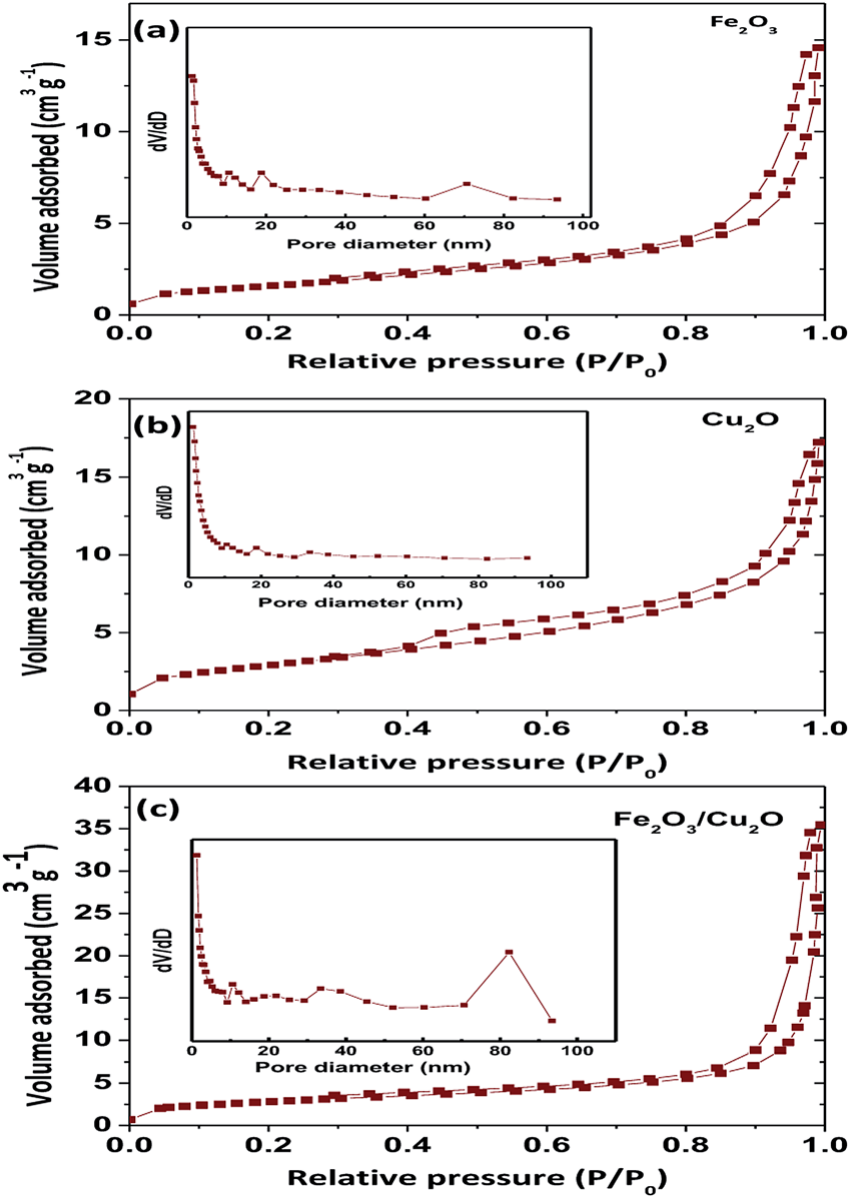
BET, N_2_ adsorption isotherms of (a) Fe_2_O_3_, (b) Cu_2_O, and (c) Fe_2_O_3_/Cu_2_O.

Surface area is an important parameter to determine the photocatalytic activity of the nanoparticles. A photocatalyst with a high surface area is likely to absorb more dye molecules and react faster (Table 1); the BET surface area as a function of the pore volume of the prepared samples undoubtedly demonstrates a similar type II curve. The BET surface area of Fe_2_O_3_, Cu_2_O and Fe_2_O_3_/Cu_2_O were found to be 5.676, 9.90, and 10.401 m^2^ g^−1^, respectively (Fig. 6). According to the hysteresis loop in the relative pressure region around 0.4–0.9, the nitrogen adsorption/desorption isotherms showed that the Fe_2_O_3_/Cu_2_O exhibited a similar type IV curve. In other words, the Fe_2_O_3_/Cu_2_O nanocomposite existed with a mesoporous structure. The surface area of the Fe_2_O_3_/Cu_2_O repressed a huge difference in the bare nanoporous Fe_2_O_3_ and Cu_2_O. With an increase in the surface area and decrease in pore size, as well as the large total pore volume, the Fe_2_O_3_/Cu_2_O is expected to have a high photocatalytic activity.

**Table 1:** Surface parameters of Fe_2_O_3_, Cu_2_O and Fe_2_O_3_/Cu_2_O

Photocatalyst	*S*_BET_ (m^2^ g^−1^)	Pore volume (cm^3^ g^−1^)
Fe_2_O_3_	5.676	1.304
Cu_2_O	9.90	2.275
Fe_2_O_3_/Cu_2_O	10.401	2.389

The common Tauc approach was used to approximate the band gap energy (*E*_g_) values. It was found that the band gap energy values of Fe_2_O_3_, Cu_2_O and the Fe_2_O_3_/Cu_2_O nanocomposite were respectively 1.96 eV, 1.89 eV and 1.85 eV. The surface charge on the metal oxides in aqueous solution becomes obvious: recognition to the progression of hydration, protonation, and deprotonation of the surface groups.^45^ RB is a cationic dye.^46^ JG is basic dyes which, together with (RB), are scarcely absorbed on the Fe_2_O_3_/Cu_2_O surface due to its negative zeta potential. However, the deliberate zeta potential was found to be −46.2 mV, suggesting the presence of a negative charge on the surface of the Fe_2_O_3_/Cu_2_O photocatalyst.^47^ The negative surface charge favours the adsorption of cationic as well as basic dyes due to the increased electrostatic force of attraction. The surface conductivity of the Fe_2_O_3_/Cu_2_O photocatalyst was found to be 0.229 mS cm^−1^.

**Fig. 7:**
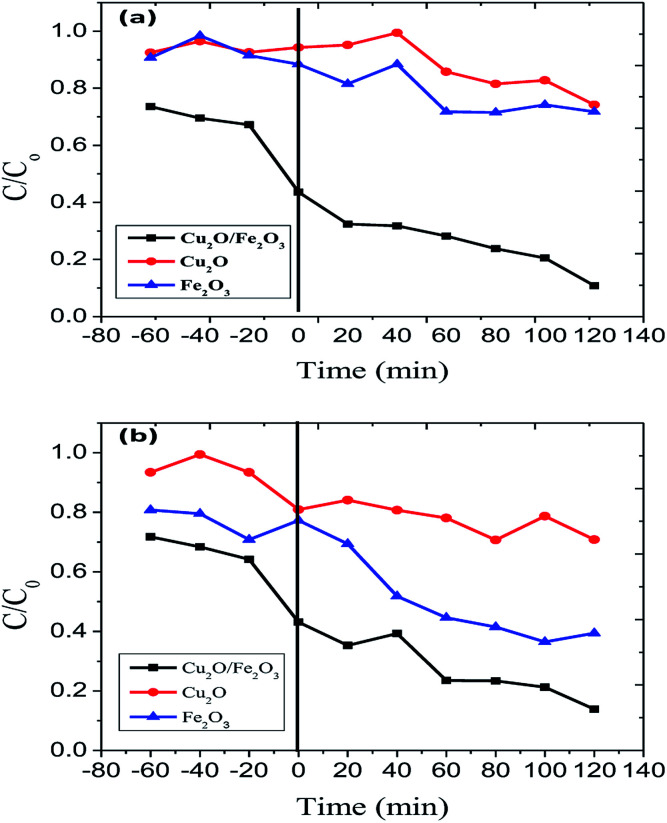
Photo-removal of (a) Rhodamine-B (RB) and (b) Janus green (JG) using Cu_2_O, Fe_2_O_3_ and the Fe_2_O_3_/Cu_2_O photocatalysts.

The surface properties of Fe_2_O_3_, Cu_2_O were affected by changes in pH. The pzc (point of zero charge) values for Fe_2_O_3_ and Fe_2_O_3_, Cu_2_O were found to be 6.9 and 7.2 respectively. It was observed that RB and JG in an acidic environment can advantageously increase the electrostatic attraction between the protons from the catalysts, including the dyes, RB and JG. Thus, photo-removal activity is high. At low pH (below 5), the chances for agglomeration are high, thus reducing the active surface area available for dye adsorption and photon absorption. At an optimum pH, the predominant iron site, namely Fe(OH)^2+^, not only forms Fe(ii), the major catalytic species in the photo-removal reactions, but also produces additional ^−^OH responsible for dye removal. When the pH value was greater than pzc, the surface of Fe_2_O_3_/Cu_2_O became negatively charged. Therefore, the negatively charged dye molecules were repelled by the catalyst surface, leading to a decrease in the effective photocatalytic activity. Highly alkaline conditions are favourable for the generation of a large number of less reactive high-valence composite iron species (Fig. 7).

**Fig. 8:**
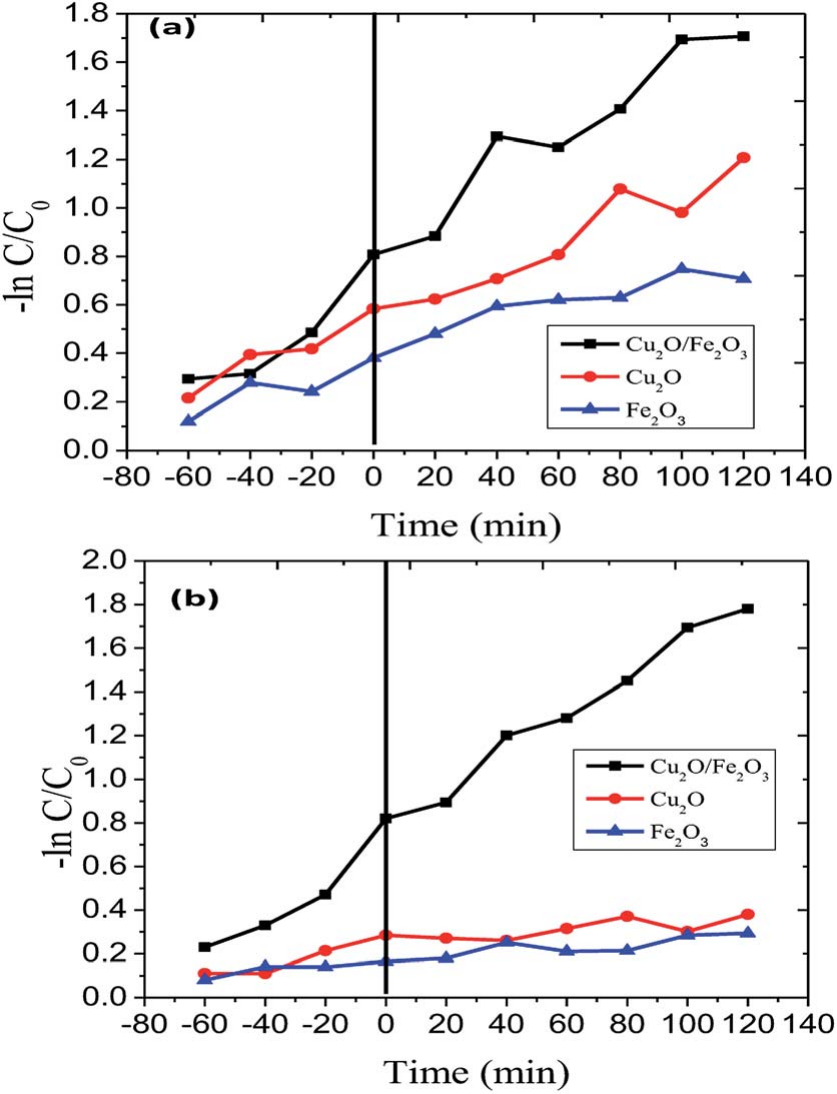
Kinetics of (a) Rhodamine-B (RB) and (b) Janus green (JG) photo-removal using Cu_2_O, Fe_2_O_3_ and the Fe_2_O_3_/Cu_2_O photocatalyst.

The Langmuir–Hinshelwood model was engaged to investigate the kinetics of RB and JG photo-removal. The photocatalytic experiments were carried out under optimal reaction conditions [Fe_2_O_3_ = 0.75 g l^−1^, both RB and JG = 9 mM and pH 5]. Fig. 8 shows the logarithmic plot of RB and JG concentration as a function of irradiation time. The photocatalytic removal of RB and JG follows pseudo-first-order kinetics; the pragmatic rate constant for Fe_2_O_3_/Cu_2_O of 1.21 × 10^−2^ s^−1^ is significantly higher than those of Fe_2_O_3_ (4.36 × 10^−3^ s^−1^) and CuO_2_ (6.45 × 10^−3^ s^−1^). Hence, the activity of the Fe_2_O_3_ nano-porous material is about approximately 2.6 times higher than that of other demonstrated materials in a systematic manner. DRS was used to investigate the light-harvesting nature of the Fe_2_O_3_/Cu_2_O photocatalyst. Estimating the conduction-band minimum (CBM) and the valence-band maximum (VBM) is vital to understanding the mechanism of the photocatalytic degradation of the photocatalyst. To investigate the CBM and VBM of Fe_2_O_3_, Cu_2_O, or Fe_2_O_3_/Cu_2_O, the UV-DRS spectra were used to record the spectrum. As shown, the associated band gap values were calculated using the following eqn:^48^*αhν* = *A*(*hν* − *E*_g_)^*n*^

**Fig. 9:**
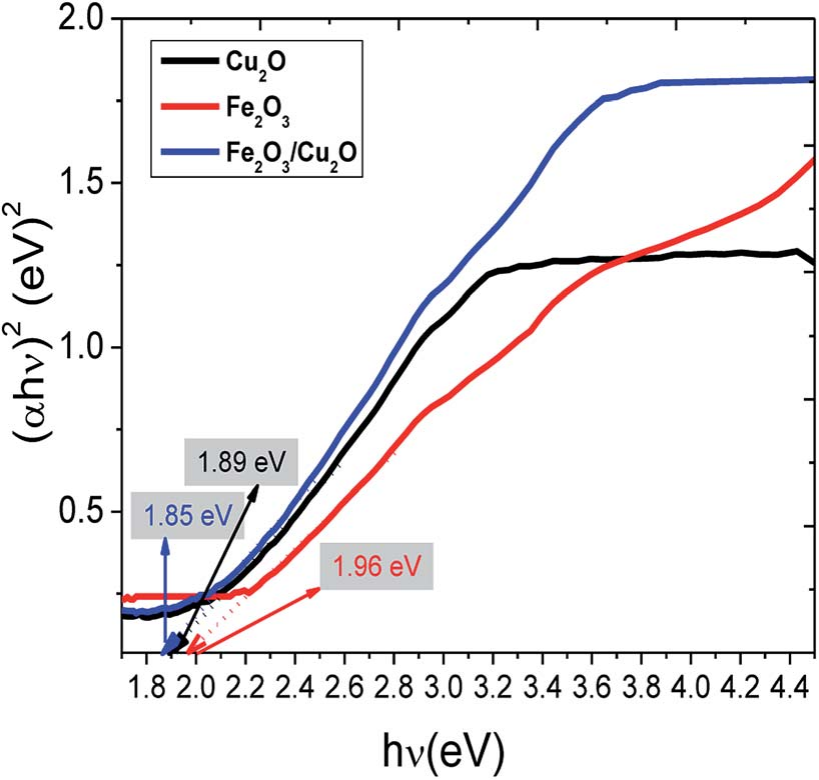
The band gap calculation using the Tauc plot of pristine Fe_2_O_3_, Cu_2_O and the Fe_2_O_3_/Cu_2_O photocatalyst.

The calculated band gaps of bare Fe_2_O_3_ and Cu_2_O were found to be 1.96 and 1.89 eV, (Fig. 9) respectively, which are consistent with similar results obtained from related work.^49^ The observed small red-shift in the band gap value (1.85 eV) of the Fe_2_O_3_/Cu_2_O photocatalyst is possibly due to the formation of a p–n heterojunction between the p-type Cu_2_O and the n-type Fe_2_O_3_.^50^

In the exploration of the photocatalytic dye removal activity, it was found that the 1.5% loading of Fe_2_O_3_ on Cu_2_O exhibits sophisticated performance compared to those of 0.8 and 2.4% loading. Fig. 7 displays the rate of the adsorption and photo-removal of Rhodamine-B and Janus green dyes for Cu_2_O, Fe_2_O_3_ and Fe_2_O_3_/Cu_2_O. Due to its negative surface charge, the optimized photocatalyst Fe_2_O_3_/Cu_2_O was found to have adsorbed the dyes under visible-light irradiation. The Fe_2_O_3_/Cu_2_O photocatalyst had effectively degraded the RB and JG dyes. It can be clearly seen that 1.5% Fe_2_O_3_ loaded onto the Cu_2_O photocatalyst is much more efficient than bare Cu_2_O towards cationic as well as basic dyes like RB and JG removal. Compared to bare Fe_2_O_3_ or Cu_2_O, Fe_2_O_3_/Cu_2_O was a much more effective photocatalyst that enhances the removal rate of Rhodamine-B and Janus green.

### 3.2. Total organic carbon (TOC) and chemical oxygen demand (COD) studies of Rhodamine-B (RB-B) and Janus green (JG)

**Fig. 10:**
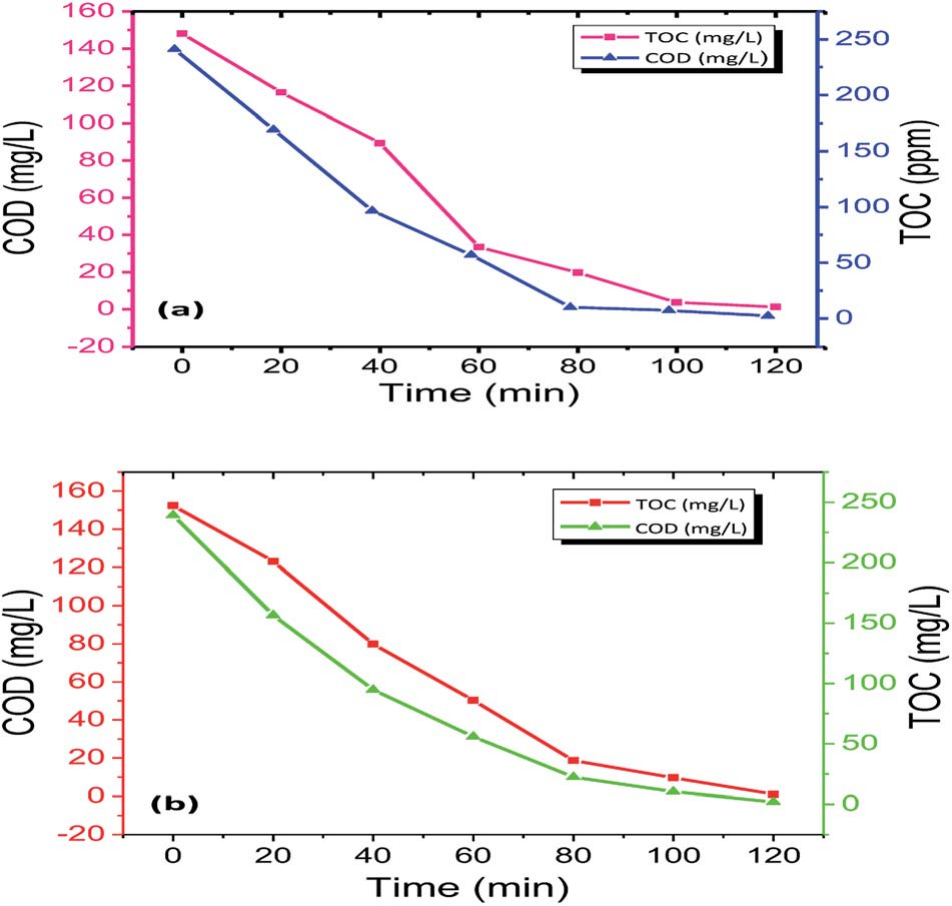
TOC and COD measurements of (a) Rhodamine-B (RB-B) and (b) Janus green (JG) degradation by the Fe_2_O_3_/Cu_2_O photocatalyst.

This analysis is necessary because the disappearance of dye colour alone cannot be used as a measure to determine the complete mineralization of the dyes.^51–54^ Noxious and long-lasting reaction intermediates were formed during the photo-removal of the dyes. Simultaneously, it was essential to calculate the degree of degradation of Rhodamine-B (RB) and Janus green (JG) during photo-removal using the Fe_2_O_3_/Cu_2_O photocatalyst. After the degradation process, it was a necessity to measure the chemical oxygen demand (COD) and total organic carbon (TOC) to assess the purity of degraded dyes before the discharging process. Total organic carbon (TOC) analysis was performed in order to determine the extent of mineralization of Rhodamine-B (RB-B) and Janus green (JG) during the photocatalytic degradation process. The present work shows the significantly declining performance in the particular functioned period (Fig. 10). Furthermore, the photocatalytic degradation process can result in the formation of colourless dye intermediates resulting in the disappearance of colour, which may actually be more toxic than the dye itself. The present study revealed that the colour disappearance of the dye was faster than the degree of mineralization with maximum TOC removal (Table S3 in ESI†). The rapid loss of colour might arise from the cleavage of the azo bond, while the high TOC value may be due to difficulty in converting the N-atom of the dye into oxidized nitrogen compounds. This mechanism clearly explained that the dye molecules were converted to other intermediates and that the dye was systematically decolourized in 120 min, which may lead to complete mineralization. Similarly, the reduction of COD reflects the extent of removal or mineralization of an organic species (Table S4 in ESI†), the percentage change in COD and TOC during photo-removal was measured under optimum reaction conditions [Rhodamine-B (RB) and Janus green (JG) concentration 9 mM, catalyst concentration 0.75 g l^−1^, pH = 5 and irradiation time of up to 120 min]. The solutions obtained after 120 min of photo-removal showed a significant decrease in COD and TOC concentration. It has been observed that Rhodamine-B (RB) and Janus green (JG) molecules were partially degraded to intermediates, and only a small fraction was subjected to complete mineralization; the COD showed a related emergent action similar to TOC.

### 3.3. Photodegradation intermediates of Rhodamine-B (RB-B) and Janus green (JG)

**Fig. 11:**
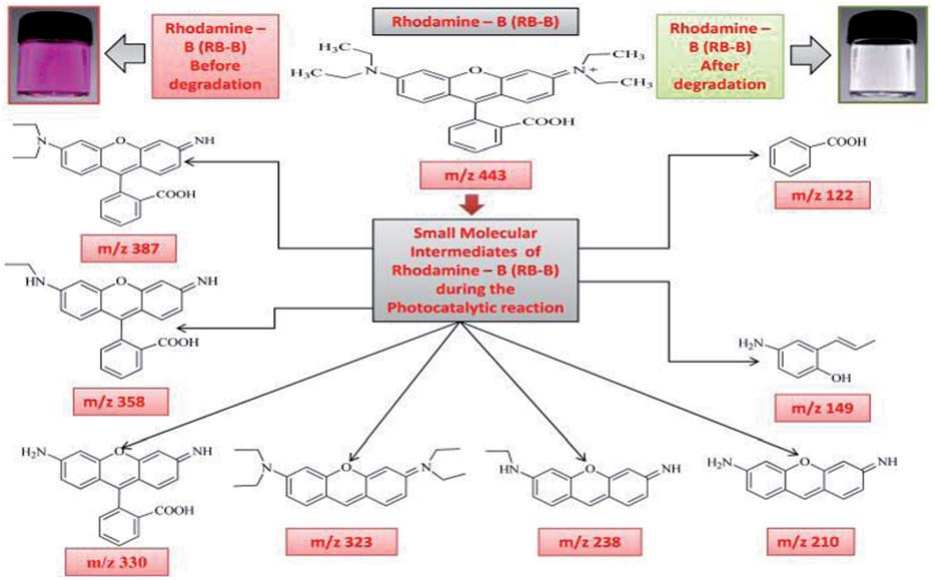
Photodegradation intermediates of Rhodamine-B (RB-B).

**Fig. 12:**
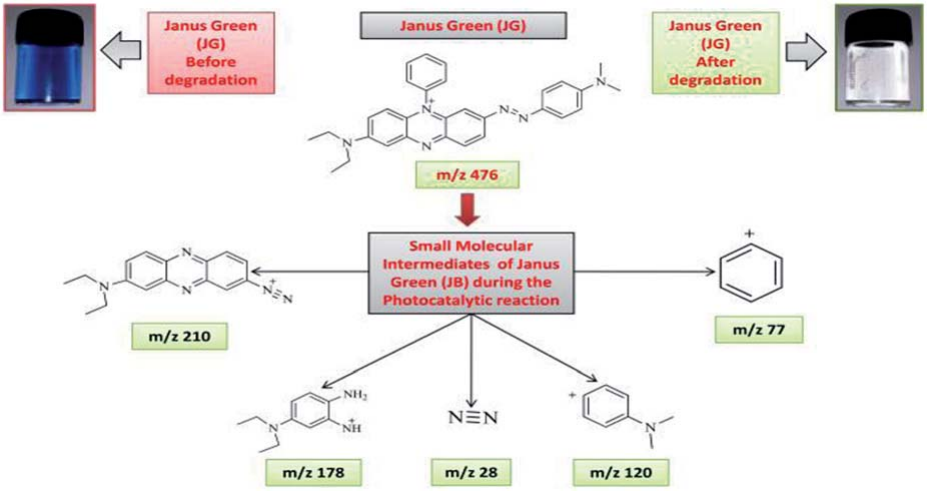
Photo-degradation intermediates of Janus green (JG).

The intermediates and final products would help to figure out the details of the reaction process. Because of the spectral overlap between the original dye and its degradation intermediates. Temporal variations during the photooxidation of Rhodamine-B (RB) and Janus green (JG) were systematically executed by LC-MS. According to earlier reports,^55–57^ small molecules were mineralized to form CO_2_ and H_2_O, which can be proven by the results of the TOC measurement. The two competitive processes occurred simultaneously during the photoreaction: *N*-deethylation and destruction of dye chromophore structure, under visible light irradiation. Most of the *N*-deethylation processes were preceded by the formation of a nitrogen-centered radical, while the destruction of dye chromophore structures is preceded by the generation of a carbon-centered radical. The photogenerated active species such as ·OH could directly attack the central carbon of Rhodamine-B (RB) (Fig. S4 in ESI†) and Janus green (JG) (Fig. S18 in ESI†), for the degradation of the dye. These dynamic species work on any *N*-deethylation intermediates to continue the deethylation process and turn the adsorption of Rhodamine-B (RB) and Janus green (JG) on the Fe_2_O_3_/Cu_2_O catalyst surface. Intermediates of the photocatalytic degradation of Rhodamine-B (RB) (Fig. 11) and (Fig. S5–S15 in ESI†), using Fe_2_O_3_/Cu_2_O are benzoic acid, benzylidyneoxonium, (*E*)-4-amino-2-(prop-1-en-1-yl)phenol, 3-imino-3*H*-xanthen-6-amine, (*Z*)-3-(ethylimino)-3*H*-xanthen-6-amine, (*E*)-*N*,*N*-diethyl-3-(diethylammonium)-3*H*-xanthen-6-amine, 2-(6-amino-3-imino-3*H*-xanthen-9-yl)benzoic acid, 2-(6-(ethylamino)-3-imino-3*H*-xanthen-9-yl)benzoic acid and 2-(6-(diethylamino)-3-imino-3*H*-xanthen-9-yl) benzoic acid, as clearly demonstrated in (Table S1 in ESI†). In Fig. 12, S16 and S17 in ESI,† photocatalytic degradation of Janus green (JG) resulted in the evolution of nitrogen, which took prominence over deethylation, leading to the formation of dinitrogen, benzene-1-ylium, 4-(dimethylamino)benzene-1-ylium, *N*_4_,*N*_4_-diethylbenzene-1,2,4-triamine cation, 8(diethylamino) phenazine-2-diazonium intermediates (Table S2 in ESI†). In the present context, the cleavage of the dye chromophores by Fe_2_O_3_/Cu_2_O has been hypothesized.

**Fig. 13:**
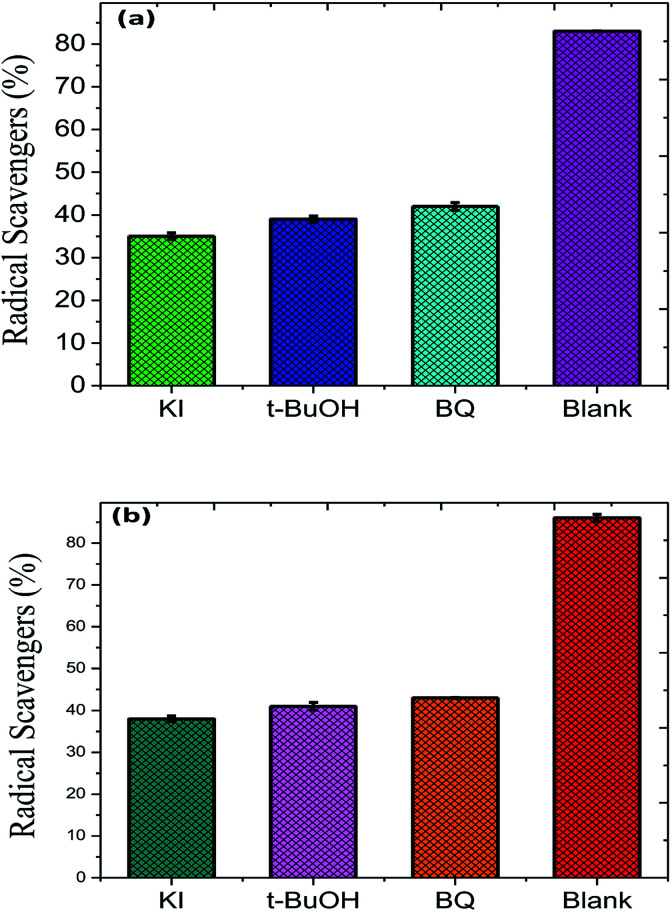
Role of different radical scavengers on the photo-removal of (a) Rhodamine-B (RB) and (b) Janus green (JG).

To evaluate the stability and re-usability of the Fe_2_O_3_/Cu_2_O photo-catalyst, five additional cycles of dye RB and JG removal were performed with it. Fig. 13 shows the good recyclability of the Fe_2_O_3_/Cu_2_O photo-catalyst for five consecutive cycles. A slight decrease in activity for the fifth cycle of RB and JG may be due to the loss of catalyst due to its recyclability. This result clearly indicates that both dyes degraded into carbon dioxide and water as the final products. The experiments were carried out under optimal reaction conditions (RB and JG = 9 mM, Fe_2_O_3_, Cu_2_O and Fe_2_O_3_/Cu_2_O = 0.75 g l^−1^ and irradiation time = 120 min) in the presence of scavengers (2 mM for 200 ml of respective dye solution) such as *t*-BuOH for ·OH,^58^ benzoquinone (BQ) for O^2^˙,^59^ and potassium iodide (KI) for holes and ·OH.^60^ The effect of *t*-BuOH, BQ and KI on the photo-removal percentage of RB and JG is shown in (Fig. 6). It was clearly observed that the photo removal percentage of RB was reduced to 35.79%, 39.78%, 43.11% and 91.25% after the addition of KI, *t*-BuOH, BQ and blank, respectively. Then again, the Janus green (JG) is as follows: *t*-BuOH-43.65%, BQ-44.78%, KI-38.65%, and blank-89.14%. The photocatalytic activity of the nanomaterial surprisingly concealed the scavenge ring effect, indicating that both O^2^˙ and the ·OH are actively implicated in the photo-removal process.

**Fig. 14:**
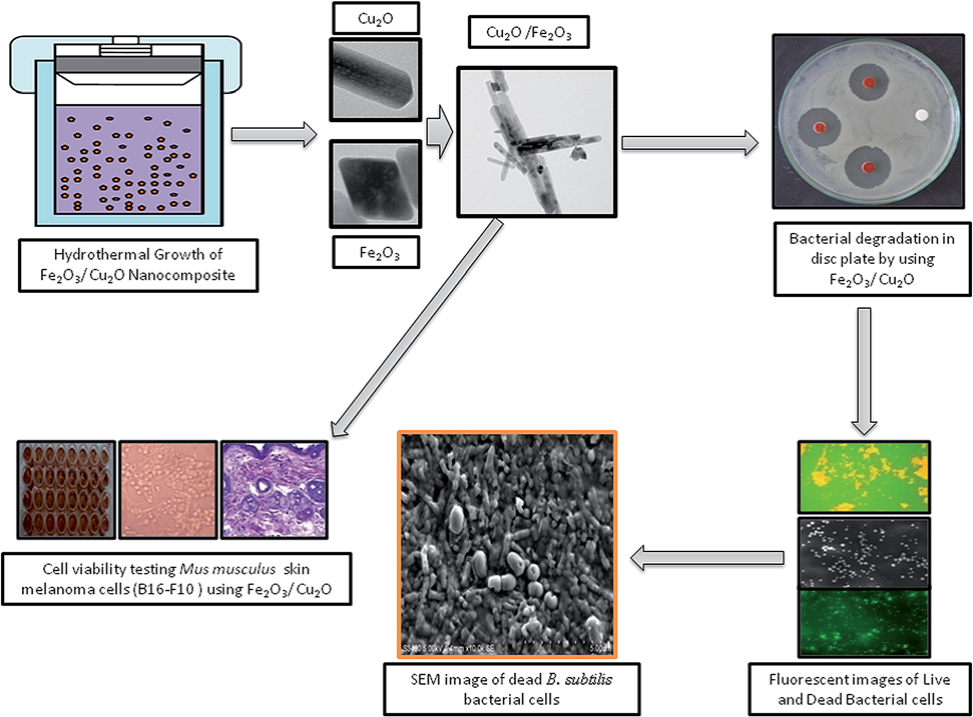
Hydrothermal growth of the Fe_2_O_3_/Cu_2_O nanocomposites and the bacterial degradation and cell viability testing studies.

### 3.4. Determination of the antibacterial activity of Cu_2_O, Fe_2_O_3_ and the Fe_2_O_3_/Cu_2_O photocatalyst nanocomposite

**Fig. 15:**
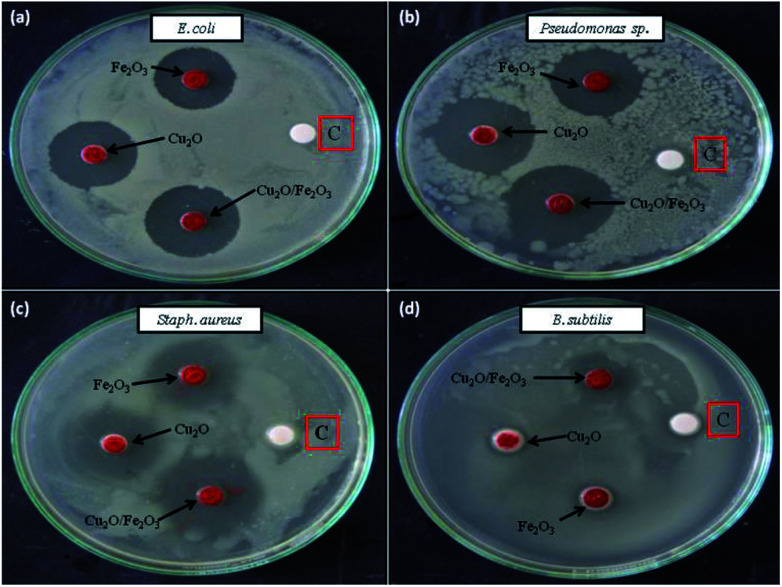
Antibacterial activity of different Fe_2_O_3_, Cu_2_O and Fe_2_O_3_/Cu_2_O nano-composites (a) *E. coli*; (b) *P. aeruginosa*; (c) *Staph. aureus*; (d) *B. subtilis*; [C] sterile distilled water.

**Fig. 16:**
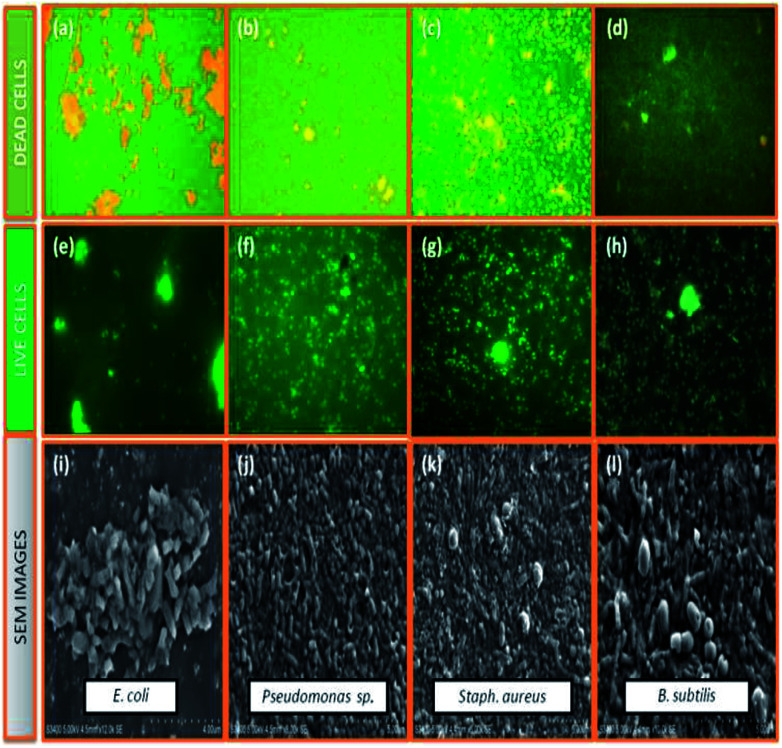
Selected live and dead cell representative fluorescent microscopic images (40 X) and scanning electron microscopic images of dead cells, (a, e and i) *E. coli*, Fe_2_O_3_; (b, f and j) *Pseudomonas* sp., Cu_2_O; (c, g and k) *Staph. aureus*, Fe_2_O_3_/Cu_2_O; (d, h and i) *B. subtilis*, Fe_2_O_3_/Cu_2_O in-general both of the series; green dots represent live bacterial cells, and yellow/orange dots represent dead cells.

Cu_2_O, Fe_2_O_3_ and the Fe_2_O_3_/Cu_2_O were further tested for their potential to inhibit test bacterial pathogens by the disc diffusion method (Fig. 15). The result of antibacterial activity showed that there exists a significant zone of inhibition against test pathogens (Table 3). It should be noted that, among the test samples, due to its large surface area, Fe_2_O_3_/Cu_2_O composites respectively yields the maximum inhibition zones of 20.13, 21.09, 08.23 and 20.60 for *Staph. aureus*, *P. aeruginosa*, *B. subtilis* and *E. coli*. In the case of *B. subtilis*, whilst only a slight response to Fe_2_O_3_/Cu_2_O nanocomposite is observed, we have not observed any zone of inhibition in SDW (Sterile Distilled Water) diffused discs against test pathogens. This study clearly suggests that the Fe_2_O_3_/Cu_2_O nanocomposite inhibits bacterial pathogens by rupturing the outer and inner walls of the cell, which leads to disorganization and leakage of the cell membrane (Fig. 16).

### 3.5. Probable photocatalytic mechanism with Fe_2_O_3_/Cu_2_O as the photocatalyst

**Fig. 17:**
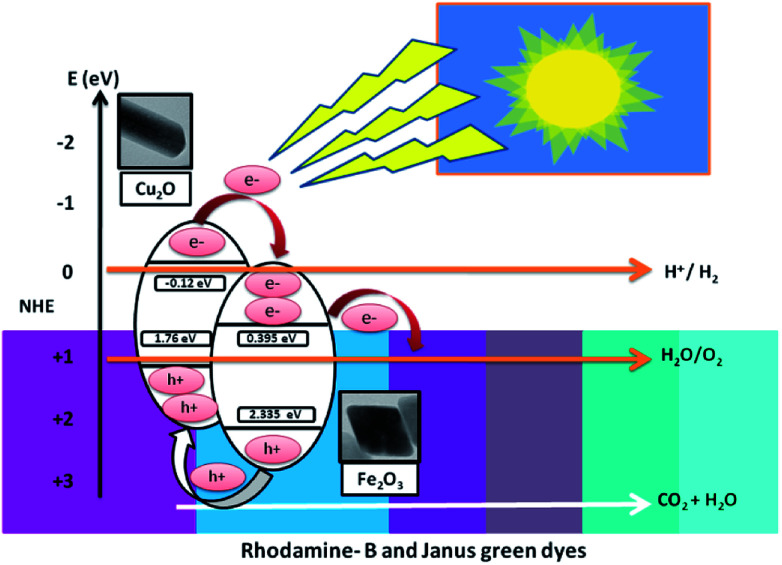
Projected photocatalytic removal mechanism under visible light irradiation.

This section clearly explains how the movement of the photo-induced charge carriers occurring between Fe_2_O_3_ and Cu_2_O have been systematically estimated using Mulliken-electronegativity theory.^61^ This theory helps explain how a p–n heterojunction functions, according to Anderson's model.^62,63^ The Mulliken-electronegativity of the semiconductor, *E*_CB_ and *E*_VB_, are respectively the conduction and valence band edge potential, where the band gap of the semiconductor *E*_g_ and *E*_e_ is the free energy of electrons on the H_2_ (4.5 eV), for Cu_2_O and Fe_2_O_3_. The electronegativity values were reported to be 5.32 and 5.88 eV, respectively. The probable band gap values of Cu_2_O and Fe_2_O_3_ from the UV-Vis spectrum determine the diffuse reflectance. The conduction and the valence band edges of the Fe_2_O_3_/Cu_2_O photocatalyst are given in Table 2. Both the conduction band edge of Cu_2_O and Fe_2_O_3_ are respectively negative and positive with respect to the hydrogen reduction potential on the normalized hydrogen scale (Fig. 17). Likewise, the valence band edge of Fe_2_O_3_ is more positive than that of Cu_2_O. Both semiconductors with different electronegativities and bands positions in an internal electric field at either side of the junction will build up, being directed from the Fe_2_O_3_ surface to the Cu_2_O surface and become exposed to visible spectrum (400 nm). The photo-generated electrons will, under the influence of an inner electric field, shift from p-type Cu_2_O to n-type Fe_2_O_3_, and the photo-created holes force to transfer from the valence bands of Fe_2_O_3_ to the valence band of Cu_2_O. These processes in effect separate and mobilize the photo-generated electron and holes, thereby enhancing the photocatalytic activity of the Fe_2_O_3_/Cu_2_O photocatalysts.

**Table 2:** The electronegativity, band gap, conduction band (CB) edge and valence band (VB) edge potential of the catalysts on a normalized hydrogen scale

Semiconductor catalyst	*x* (eV)	*E*_g_ (eV)	*E*_CB_ (eV)	*E*_VB_ (eV)
Fe_2_O_3_	5.88	1.96	−0.12	2.355
Cu_2_O	5.32	1.88	0.395	1.76

**Table 3:** Antibacterial activity of the Fe_2_O_3_/Cu_2_O nano-composite against test pathogens^a^

Test sample	Zone of inhibition (in mm)			
	*B. subtilis*	*Staph. aureus*	*P. aeruginosa*	*E. coli*
Fe_2_O_3_	04.53 ± 0.11	15.21 ± 0.33	17.24 ± 0.12	17.41 ± 0.06
Cu_2_O	02.22 ± 0.24	16.24 ± 0.33	17.10 ± 0.23	18.17 ± 0.07
Fe_2_O_3_/Cu_2_O	08.23 ± 0.14	20.13 ± 0.11	21.09 ± 0.32	20.60 ± 0.11
Std. DW	00.00 ± 0.00	00.00 ± 0.00	00.00 ± 0.00	00.00 ± 0.00

a Values are means of three independent replicates; ± indicate standard error.

### 3.6. Photo-removal mechanism of dyes

Hydrogen generation from photo-induced water break-down is increasingly seen as a feasible choice to concurrently solve energy and environmental problems. Various photo-induced H_2_ generation techniques and photocatalytic water splitting was demonstrated in the visible light spectrum.^64–68^ The low cost and high sustainability features of the reaction system in the photo-removal mechanism of dyes are explained in S1 in ESI.†

Electron–hole pairs in the excited Fe_2_O_3_ could be efficiently separated to facilitate an efficient shift into photo-induced electrons between Fe_2_O_3_ and Cu_2_O. This radical splitting process makes a crucial task in the removal of dyes. The effectiveness of the photo-reaction very much depends on the efficiency of the adsorption of untreated organic contaminants on the photocatalysts and the process of splitting photo-created electron–hole pairs. The holes can either react or be adsorbed through surface hydroxyl to form hydroxyl radicals. As a result, the adsorption equilibrium was destroyed, permitting dye molecules to move from single elucidation to the interface and to consequently decompose into CO_2_, H_2_O and other raw materials through redox reactions.

### 3.7. Re-usability of photocatalysts

**Fig. 18:**
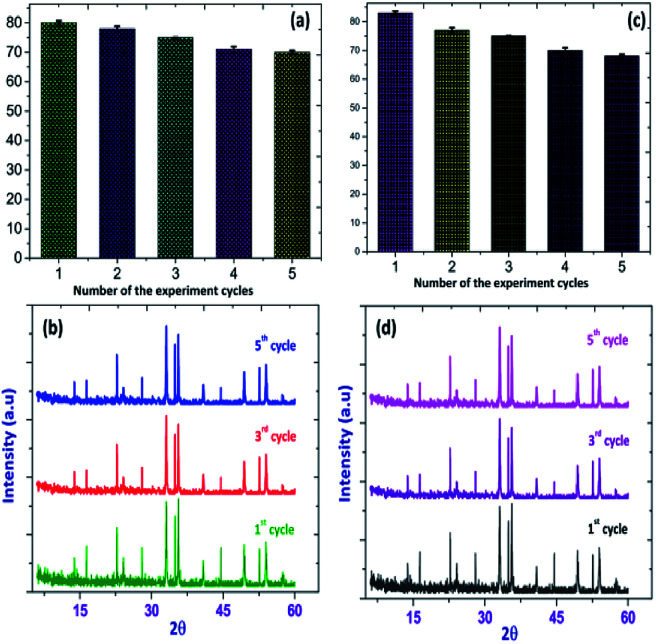
Recycling performance of the photo-removal of (a) Rhodamine-B (RB) (b) Janus green (JG), and XRD patterns of (c) Rhodamine-B (RB): Fe_2_O_3_/Cu_2_O (d) Janus green (JG): Fe_2_O_3_/Cu_2_O before and after successful cycles of recycling.

The sustainability of a photocatalyst is the most important concern for the booming industry. In order to investigate the stability and durability of Fe_2_O_3_/Cu_2_O, nanomaterial recycling experiments were conducted for the photo-removal of RB and JG. After the completion of each cycle, the photocatalyst was collected using an external magnet, washed with double distilled water, dried overnight, and reused.^66,67^ The photo-removal percentages of RB and JG for five successive cycles were found to be 79.15%, 74.65%, 75.45%, 74.98% and 73.24%, respectively, and are shown in Fig. 18.

The Fe_2_O_3_/Cu_2_O nanomaterial exhibits an average of 76.24% for photocatalytic activity after five successive cycles. The reduction in activity after two cycles is due to the loss of catalyst during the washing process.^69^ In addition, there is no obvious change observed in the XRD pattern of Fe_2_O_3_ in (Fig. 18) after five cycles. These results indicated that the Fe_2_O_3_/Cu_2_O materials could be used as a stable photo-catalyst for the removal of organic pollutants in the form of dyes in industrial wastewater.

### 3.8. Toxicity tests of Fe_2_O_3_, Cu_2_O and Fe_2_O_3_/Cu_2_O

**Fig. 19:**
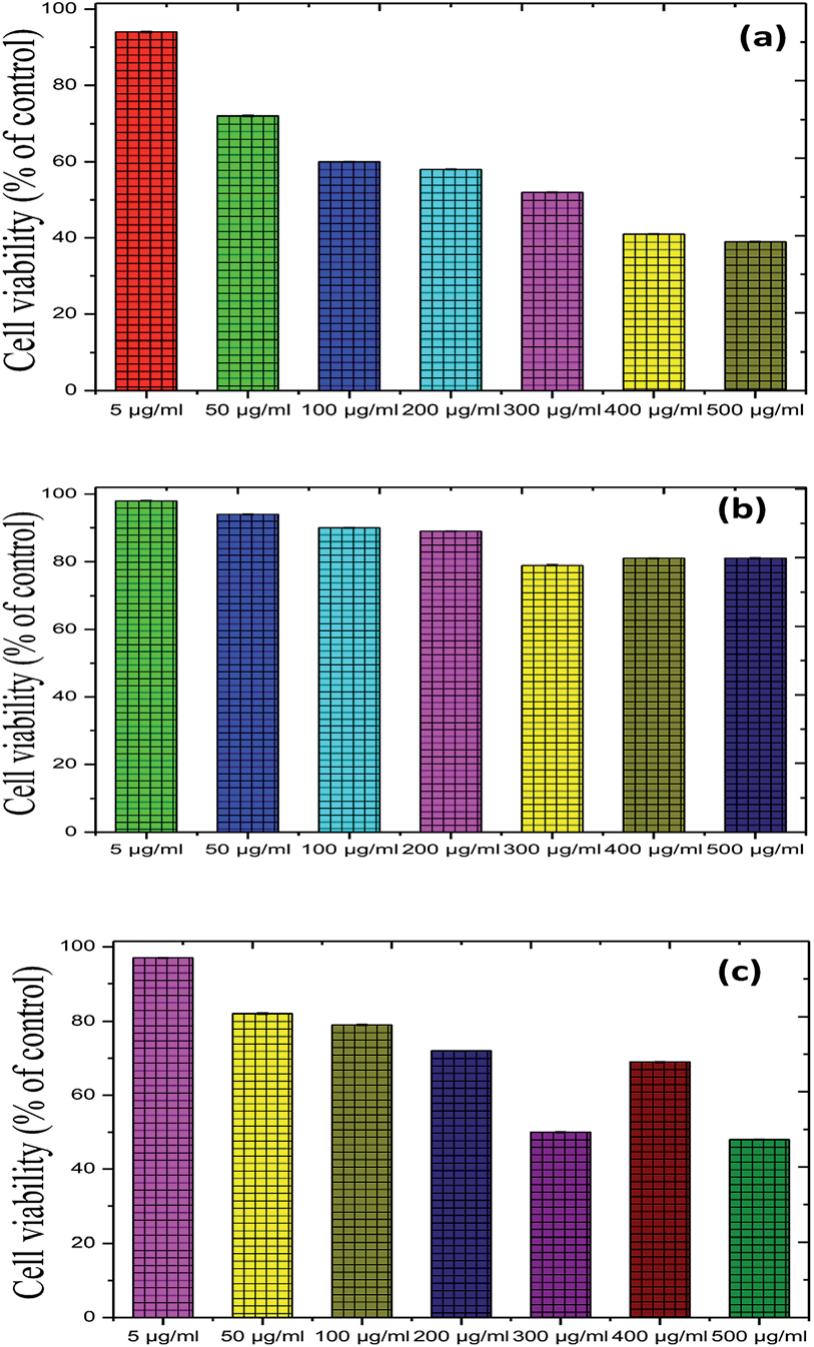
Recycling performance of the photo-removal of (a) Rhodamine-B (RB) (b) Janus green (JG), and XRD patterns of (c) Rhodamine-B (RB): Fe_2_O_3_/Cu_2_O (d) Janus green (JG): Fe_2_O_3_/Cu_2_O before and after successful cycles of recycling.

To further illustrate the bio-compatible advantage of the proposed photocatalyst, up-conversion materials for emitting visible light were used as candidates for both *in vivo* and *in vitro* bio-imaging as well as in photodynamic therapy. We measured the toxicity of Fe_2_O_3_, Cu_2_O and Fe_2_O_3_/Cu_2_O using *Musmusculus* skin melanoma cells of various concentrations (5–500 g ml^−1^). The results suggested that the viability of (B16-F10) cells decreased in a dose-dependent manner in each sample (Fig. 19), clearly showing that, at a 5 g ml^−1^ concentration, the lowest concentration of the three respective samples used in the assay did not reduce the cell viability noticeably as compared to other increased concentrations, among which Fe_2_O_3_/Cu_2_O was found to be low in a systematic manner. Therefore, Fe_2_O_3_/Cu_2_O is the safest and the superlative alternative in terms of toxicity in biological application.

## 4 Conclusions

In this experimental work, a unique and visible light-driven Fe_2_O_3_/Cu_2_O composite photo-catalyst was prepared by a simple, eco-friendly and cost-effective hydrothermal method for multi-purpose application. Rhodamine-B (RB) and Janus green (JG) could be easily decolorized by Fe_2_O_3_/Cu_2_O under visible light irradiation. Furthermore, the photocatalyst was recycled several times without any observable loss of photocatalytic activity, making it suitable for use in dye removal in wastewater. Moreover, the synthesized materials were found to be highly stable in the photocatalytic process; displayed antibacterial properties against *E. coli*, *P. aeruginosa*, *Staph. aureus* and *B. subtilis*; and anti-cancer properties against *Musmusculus* skin melanoma cells (B16-F10). These results suggest that these composite materials can be utilized in the biomedical field and for catalysis and energy conversion systems.

## Conflicts of interest

The authors declare no competing financial interests.

## Supplementary Material

RA-009-C8RA09929D-s001
