# Supplementary material for: Photocatalytic dye degradation and biological activities of the Fe2O3/Cu2O nanocomposite
Source: RSC Adv. 2019 Mar 14;9(15):8557–68. doi: 10.1039/c8ra09929d (PMC9061704; doi:10.1039/c8ra09929d)
Supplement: RA-009-C8RA09929D-s001 [file RA-009-C8RA09929D-s001.pdf]

Electronic Supplementary Material (ESI) for RSC Advances.
This journal is © The Royal Society of Chemistry 2019

RSC Advances

PAPER

Photocatalytic dye degradation and biological activities of Fe_2O_3/Cu_2O nanocomposite

Received 00th January 20xx,
Accepted 00th January 20xx

DOI: 10.1039/x0xx00000x

Mavinakere Ramesh Abhilash,*[a] Gangadhar Akshatha, [a, b], and Shivanna Srikantaswamy, [a, b]

Supplementary information

S 1: Photo-removal mechanism of dyes.

The organic dye removal by using Fe_2O_3/Cu_2O photocatalysts under an UV irradiation undergoes excitation of the photo-evaluated electrons in the conduction band and holes in the valence band. Succeeding chemical reactions take place within the media after the photo-evaluated charges became mobilized. Moreover, the dyes act as a sensitizer of visible/UV light that releases photo-excited electrons to an electron acceptor to become a cationic/anion dye free radical followed by a self-removal or removal process due to the reactive oxidation. The improved photo-removal efficiency of the Fe_2O_3, Cu_2O and Fe_2O_3/Cu_2O photocatalystsis attributed to the adsorption of contaminant molecules through catalysis, the light absorption and finally the charge separation and transportation. The optical quenching and surface resonance characteristics is higher. Fe_2O_3 valence electrons are excited to the conduction band, thereby producing the electron–hole pairs. Photo-generated electrons are transferred to Cu_2O, which acts as an electron acceptor. Cu_2O promotes systematic valence electron transfer process and effectively hinders the recombination of the photo-generated electrons and holes. The holes in the valence band of iron oxide can react with absorbed hydroxyl groups to form surface hydroxyl radicals. In the interim, the photo-generated electrons on the surface of the composite nanoparticles, as well as the trapped electrons on Cu_2O, can also react with the dissolved oxygen to form reactive oxygen species, which further react with water to form hydroxyl radicals. Both the hydroxyl radicals and holes can degrade and oxidize the dye molecule in systematic manner.

S 2: Cell culture and treatment.

The culture was maintained in a humidified environment with 5% CO_2 at 37°C (CO_2, Sanyo Incubator; Japan). In the route of the preferred meditation of Fe_2O_3, $Cu_2O$and Fe_2O_3/Cu_2O, a dose–dependent response curve with different concentrations (0.01, 0.05, 0.1, 0.5, 1.0, and 2.5µg/l) was plotted using propagation and MTT [(2-(3,5-diphenyltetrazol-2-ium-2-yl)-4,5-dimethyl1-1,3-thiazole bromide)] assay, after 72hour of exposure. For this, 105 cells were seeded in 24-well plates and cultured with fresh culture medium for 72 hour prior to treatment. Fe_2O_3, Cu_2O and Fe_2O_3/Cu_2O be diluted in di-methyl sulfoxide (DMSO; maximum 1% final concentration) and, for this basis, 1% of DMSO was added in the culture medium of the control group. The concentrations of Fe_2O_3 used in the other trails were 5, 50, 100, 200, 300, 400 and 500µg/l, and they were monitored for duration of 72 hour. All the experiments were performed in triplicate and three independent repetitions. Herein, the research test was carried out for the cell viability assay by treatment of B16-F10 melanoma cells with the prepared samples of various concentrations as mentioned above for 72 hr. Cell viability was measured using 3-(4, 5-dimethylthiazol-2-yl)- 2, 5-diphenyltetrazolium bromide (MTT) colorimetric assay.

[a]*Department of Studies in Environmental Science, University of Mysore, Manasagangotri, Mysore 570006, India*

[b]*Centre for Materials Science and Technology, Vijnana Bhavan, University of Mysore, Manasagangotri, Mysore 570006, India. E-mail: abilashmrenvi@gmail.com*

† Footnotes relating to the title and/or authors should appear here.
Electronic Supplementary Information (ESI) available: [details of any supplementary information available should be included here]. See DOI: 10.1039/x0xx00000x

Please do not adjust margins

S 3: Chemical required to synthesis of Fe_2O_3, Cu_2O and Fe_2O_3/Cu_2O nannocomposite

Ferrous chloride (99%, Sigma Aldrich), ferric chloride (97%, Sigma Aldrich), ammonium hydroxide solution (28–30%, Sigma Aldrich), hydrochloric acid (37%, Fisher Scientific), methanol (ACS reagent, Fisher Scientific), were used as received without any additional purification. Ferrous chloride, ferric chloride and ammonium hydroxide were used for the synthesis of iron oxide nanoparticles and Cupric sulphate ($CuSO_4{\cdot}5H_2O$),(99+%, Sigma Aldrich), sodium hydroxide, Dextrose anhydrous (D-Glucose) (ACS reagent, Fisher Scientific) were used for the synthesis of cupric oxide nanoparticles. All chemicals were of reagent grade and used without further purification, de-ionized water was purified by a Barnstead/Synbronnanopure-II, purification system.

S 4: Sample Characterizations and measurements.

In this study, we adopted various tools; like Braunauer–Emmett–Teller (BET), BELSORP MINI - 2, BEL, Japan, for specific surface area, pore volume, and pore size distribution of the samples were determined by N_2 sorption at 77 K using a Micromeritics ASAP-2020, Ultima III Series, RIGAKU, TSX System, Japan, used for X-ray diffraction (XRD) patterns. We employed a High Resolution Transmission Electron Microscope images of the Fe_2O_3/Cu_2O composites was captured using Jeol/JEM - 2100, magnification - 2000X – 1500000 X with 200kV acceleration voltages. The Energy Dispersive Spectroscopy (EDS) analysis was carried out using HITACHI (Noran System 7, USA) system attached to the Zeiss Supra 55VP. Fourier-transformed infrared (FT-IR) spectra were obtained on a Thermo Scientific Nicolet, 6700 Analytical FT-IR spectrometer. Zeta potential measurements of the attenuate dispersions (0.1 mg mL^{-1}) of the nanocomposites were conducted using a Brookhaven Nano-Brook Omni Instrument at 25° C. The particle size distribution (PDS) nanocomposite was monitored by using Microtrac (USA) particle size analyzer provides the size measurement, which confirms the particle size distribution. Diffuse reflectance spectrums were analyzed by a UV-visible spectrophotometer (Shimadzu UV-2550 UV-Visible spectrometer, Kyoto, Japan). The total organic carbon (TOC) concentration was determined with a TOC analyzer (TOC-5000, Shimadzu Corporation, Kyoto, Japan). COD was determined (Merck Spectroquant TR320) by a closed reflux colorimetric method. The identification of Rhodamine-B (RB) and Janus green (JG) the degradation intermediate products were measured by a liquid chromatography-mass spectrometer (Waters-USA, Synapt G-2 HDMS).

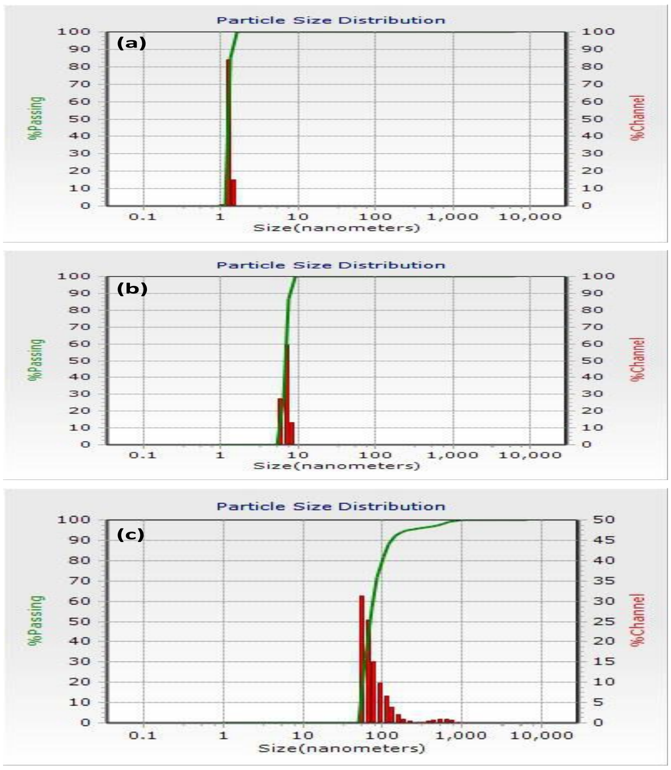

S Fig. 1 Evaluating particle size distribution and zeta potential DLS results of (a) Fe_2O_3, (b) $Cu_2O$and (c) Fe_2O_3/Cu_2O.

(a) Element	Weight (%)	Atomic (%)	Compound (%)
O K	37.98	32.22	52.24
Cu L	241.89	51.67	0.00
C	14.26	16.11	0.00
Total		294.13	

(b) Element	Weight (%)	Atomic (%)	Compound (%)
O K	197.03	51.90	270.99
Fe L	293.61	22.15	0.00
C	73.96	25.95	0.00
Total		564.60	

(c) Element	Weight (%)	Atomic (%)	Compound (%)
O K	147.03	44.60	202.22
Fe L	240.20	20.87	0.00
C	160.20	22.30	0.00
Total		602.63	

S Fig. 2 The elemental composition of Fe_2O_3/Cu_2O photo-catalyst determined from energy dispersive spectroscopy (EDS), (a) Cu_2O, (b) Fe_2O_3, (c) Cu_2O and Fe_2O_3, composites.

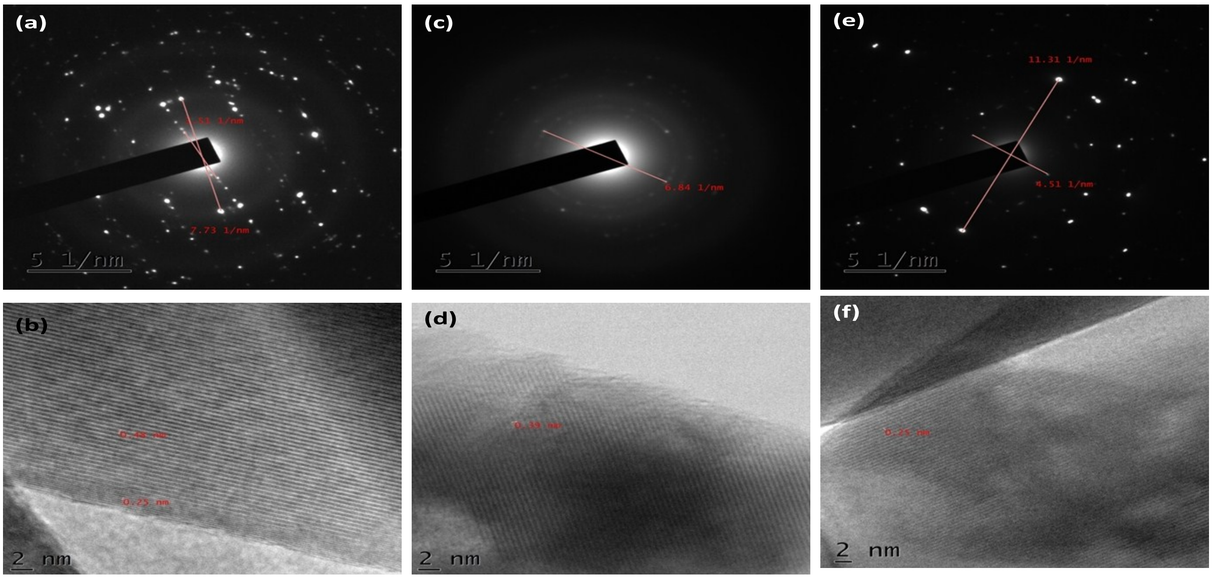

S Fig. 3 The HR-TEM, SAED pattern of (a) Fe_2O_3 (c) Cu_2O and (e) Fe_2O_3/Cu_2O composite and 2 nm images of (b) Fe_2O_3 (d) Cu_2O and (f) Fe_2O_3/Cu_2O composite

Table S 1 Photodegradation intermediates of Rhodamine – B (RB-B).

Product Code	M/Z	Chemical Formula	Name	Structure
1a	122	$C_7H_6O_2$	benzoic acid	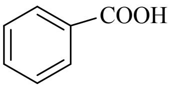
1b	105	$C_7H_5O^+$	benzylidyneoxonium	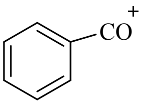
1c	149	$C_9H_{11}NO$	(E)-4-amino-2-(prop-1-en-1-yl)phenol	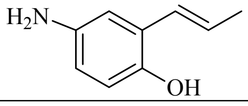
1d	210	$C_{13}H_{10}N_2O$	3-imino-3H-xanthen-6-amine	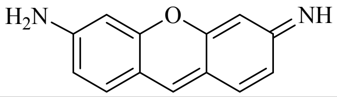
1e	238	$C_{15}H_{14}N_2O$	(Z)-3-(ethylimino)-3H-xanthen-6-amine	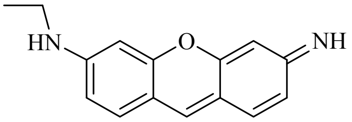
1f	323	$C_{22}H_{29}N_2O^+$	(E)-N,N-diethyl-3-(diethylimmonium)-3H-xanthen-6-amine	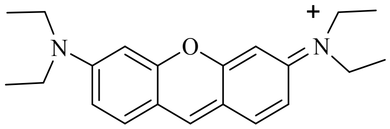
1g	330	$C_{20}H_{14}N_2O_3$	2-(6-amino-3-imino-3H-xanthen-9-yl)benzoic acid	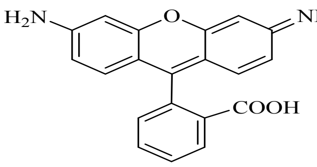

Please do not adjust margins

1h	358	$C_{22}H_{18}N_2O_3$	2-(6-(ethylamino)-3-imino-3H-xanthen-9-yl)benzoic acid	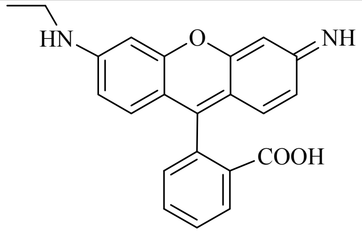
1i	387	$C_{24}H_{22}N_2O_3$	2-(6-(diethylamino)-3-imino-3H-xanthen-9-yl)benzoic acid	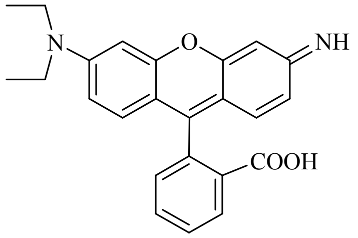

Table S2 Photodegradation intermediates of Janus green (JG).

Product Code	M/Z	Chemical Formula	Name	Structure
2a	28	N_2	Dinitrogen	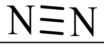
2b	77	$C_6H_5^+$	benzene-1-ylium	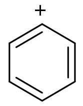
2c	120	$C_{10}H_{16}N_3^+$	4-(dimethylamino)benzene-1-ylium	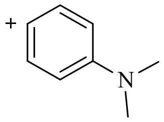
2d	178	$C_{10}H_{16}N_3^+$	N_4,N_4-diethylbenzene-1,2,4-triamine cation	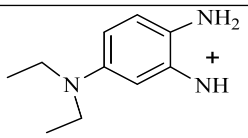
2e	210	$C_{16}H_{16}N_5^+$	8(diethylamino)phenazine-2-diazonium	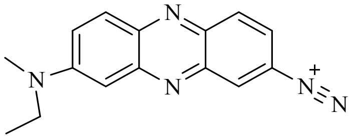

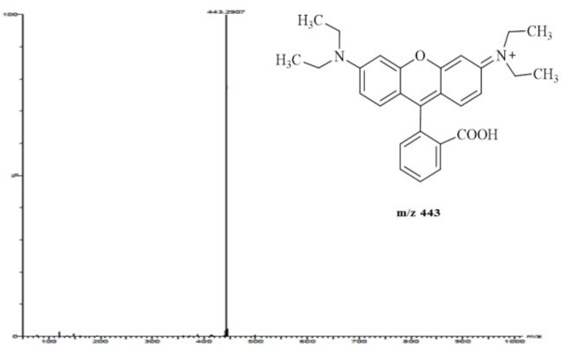

S Fig. 4 Mass spectra of Rhodamine-B (RB-B), before degradation.

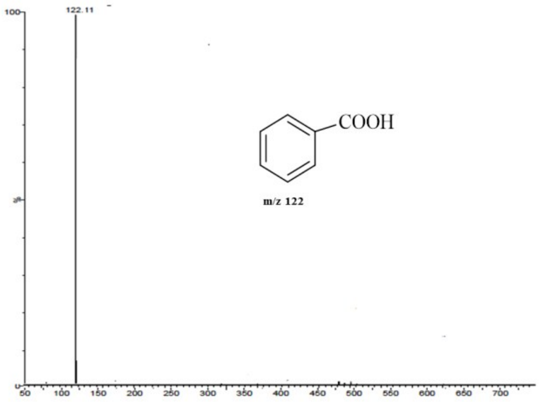

S Fig. 5 Mass spectra of benzoic acid.

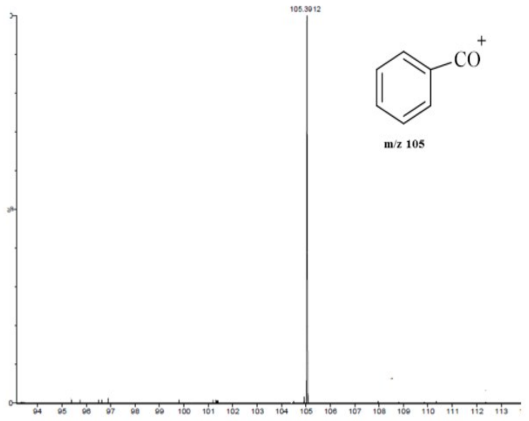

S Fig. 6 Mass spectra of benzylidyneoxonium

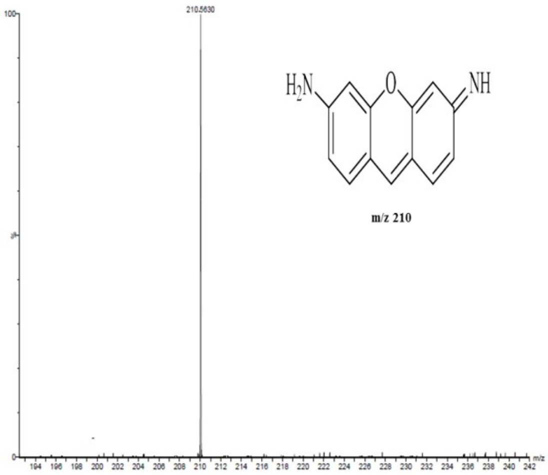

S Fig. 7 Mass spectra of 3-imino-3H-xanthen-6-amine.

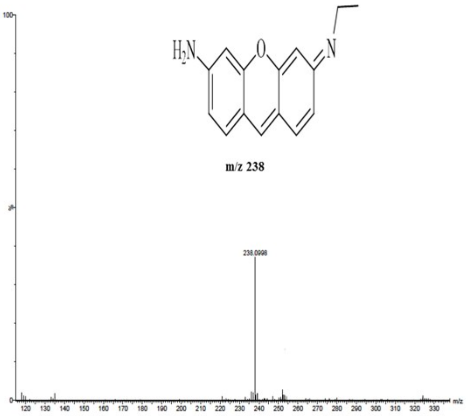

S Fig 8 Mass spectra of (Z)-3-(ethylimino)-3H-xanthen-6-amine.

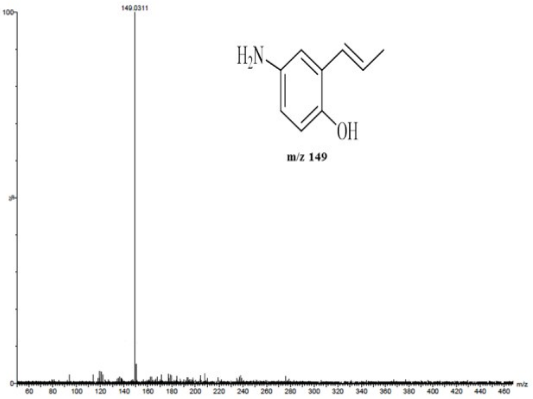

S Fig.9 Mass spectra of (E)-4-amino-2-(prop-1-en-1-yl) phenol

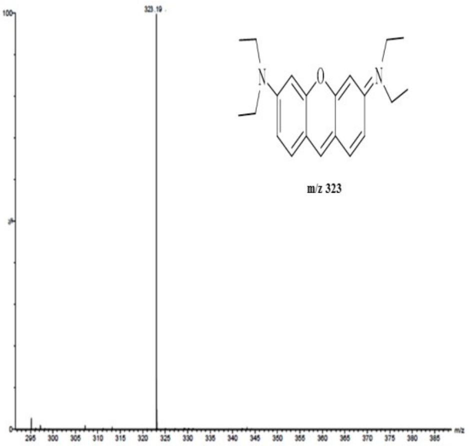

S Fig. 10 Mass spectra (E)-N,N-diethyl-3 (diethylimmonium)-3H-xanthen-6-amine

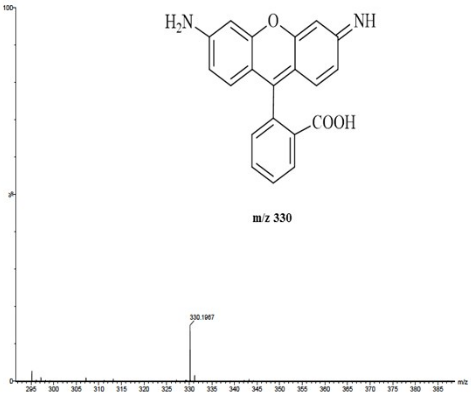

S Fig 11 Mass spectra of 2-(6-amino-3-imino-3H-xanthen-9-yl) benzoic acid

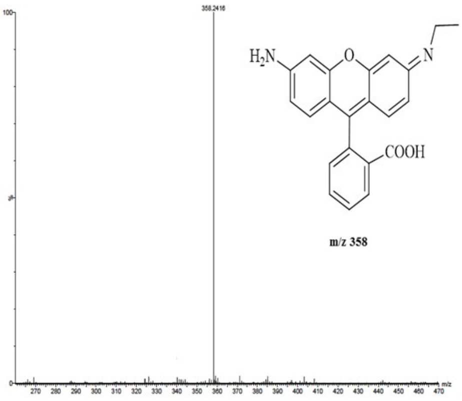

S. Fig 12 Mass spectra of 2-(6-(ethylamino)-3-imino-3H-xanthen-9-yl) benzoic acid

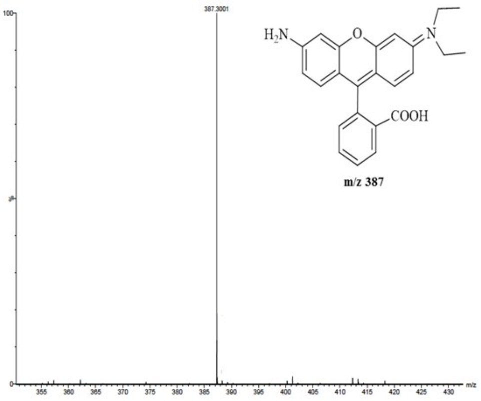

S Fig 15 Mass spectra of 2-(6-(diethylamino)-3-imino-3H-xanthen-9-yl) benzoic acid

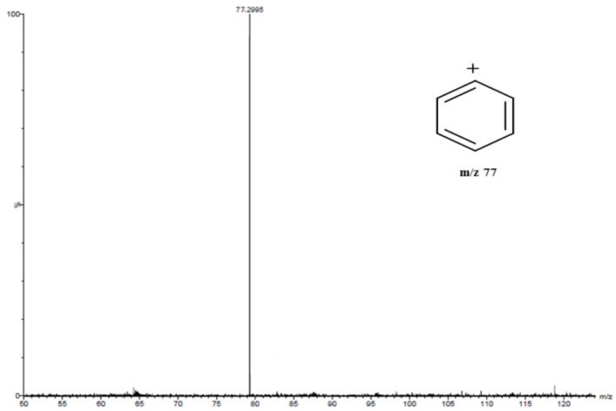

S Fig. 13 Mass spectra of benzene-1-ylium

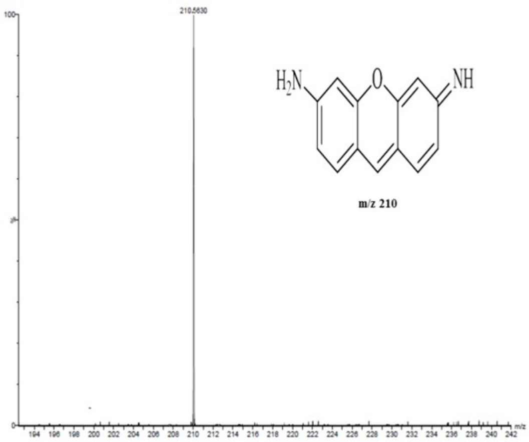

S Fig. 16 Mass spectra of 8(diethylamino) phenazine-2-diazonium

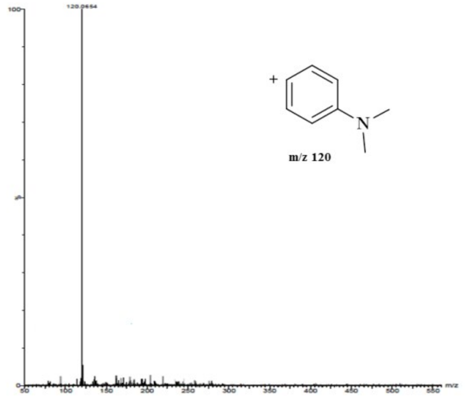

S Fig. 14 Mass spectra of 4-(dimethylamino) benzene-1-ylium.

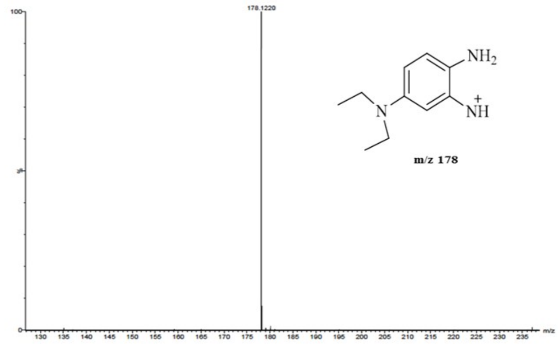

S Fig. 17 Mass spectra of N_4,N_4-diethylbenzene-1,2,4-triamine cation

Please do not adjust margins

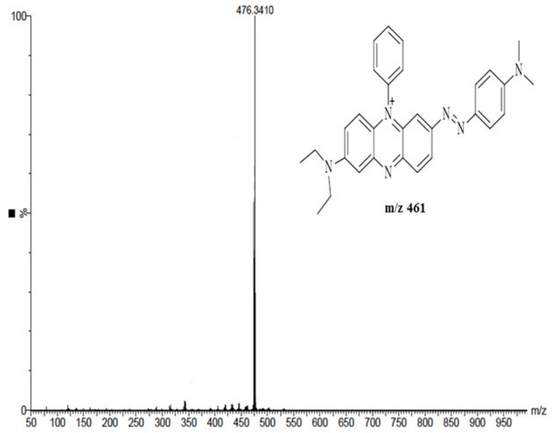

S Fig. 18 Mass spectra of Janus Green (JG), before degradation.

Table S 3 Chemical oxygen demand (COD) and Total organic carbon (TOC) of Rhodamine – B against Fe_2O_3/Cu_2O photocatalysts

Time (min)	COD (ppm)	TOC (ppm)
00	148.02	241.11
20	116.45	169.32
40	89.34	96.65
60	33.48	56.98
80	19.68	10.28
100	3.67	7.26
120	1.26	2.34

Table S 4 Chemical oxygen demand (COD) and Total organic carbon (TOC) of Janus green against Fe_2O_3/Cu_2O photocatalysts

Time (min)	COD (ppm)	TOC (ppm)
00	152.37	239.09
20	123.12	156.58
40	79.89	94.69
60	50.29	55.97
80	18.67	22.39
100	9.64	10.31
120	1.01	1.98
